# An efficient *Rhizobium rhizogenes-*mediated transformation system for *Cuscuta campestris*

**DOI:** 10.1371/journal.pone.0317347

**Published:** 2025-02-21

**Authors:** K. M. Asha Alles, P. G. L. T. Dilhani, C. H. W. M. R. Bhagya Chandrasekera, Pradeepa C. G. Bandaranayake

**Affiliations:** Agricultural Biotechnology Centre, Faculty of Agriculture, University of Peradeniya, Peradeniya, Sri Lanka; University of Kotli, PAKISTAN

## Abstract

Parasitism has evolved independently in various plant families, with *Cuscuta campestris* (field dodder) being an economically significant example. Despite advances in genomics and transcriptomics, functional studies in *C*. *campestris* are limited by the lack of an efficient genetic transformation system. This study introduces a highly effective *Rhizobium rhizogenes*-mediated transformation system for *C*. *campestris* using a pBIN plasmid harboring a Yellow Fluorescence Protein reporter gene. We optimized transformation and regeneration by assessing explant type, media composition, and plant growth regulators. Notably, host plant contact was essential for transgenic shoot regeneration. Over 70% transformation efficiency was achieved using cuttings co-incubated with modified Murashige and Skoog medium and 5 mg/L Benzylaminopurine, followed by transfer to tomato hosts. Additionally, we developed a complete *in-vivo* protocol over 30% regeneration efficiency. Transgenic shoots were confirmed for *rol* gene expression and haustoria formation, advancing functional studies in *C*. *campestris*.

## Introduction

With over 4,500 species, parasitic flowering plants rely on other plants to complete their life cycle. Within this group, the genus *Cuscuta*, commonly known as Dodder, comprises approximately 200 species, all of which are stem parasites. These *Cuscuta* species are holoparasitic, meaning they must connect with a host plant within days of germination to survive and grow [1]. They can be found in temperate and tropical regions worldwide, with species such as *Cuscuta campestris* exhibiting a broad host range [1, 2]. *Cuscuta campestris* infections often result in reduced host biomass, changes in photosynthetic pigments, and even anatomical alterations in host plants [3–5]. Due to their significant impact on crops, *Cuscuta* species were added to the US Department of Agriculture’s Noxious Weeds List in 2010.

Extensive research has positioned *Cuscuta* as a model system for studying plant-plant interactions through the haustoria, which facilitates the exchange of molecules, including DNA, RNA, and proteins, between the host and the parasite [6–9]. However, there is still limited understanding of the underlying biology and genetic regulation of *Cuscuta* parasitism, especially in functional characterizing genes and pathways. Recent advancements in genome and transcriptomic sequencing have identified candidate genes involved in parasitism, setting a foundation for further research [10–13]. High-throughput functional analysis of these genes through methods like CRISPR/Cas-based genome editing and RNA interference (RNAi) requires a stable genetic transformation system.

*Rhizobium*-mediated transformation is a prominent method for creating transgenic plants, commonly using *Rhizobium tumefaciens* (formerly *Agrobacterium tumefaciens*) and *Rhizobium rhizogenes* (formerly *Agrobacterium rhizogenes*), which facilitate stable transformation through both direct and indirect regeneration pathways [14, 15]. Existing transformation protocols, including those for hemiparasitic species like *Triphysariya versicolor* [16] and *Phtheirospermum japonicum* [17], and holoparasitic species such as *Phelipanche aegyptiaca* [18] and *Phelipanche ramose* [19], demonstrate the versatility of *Rhizobium*-mediated systems. However, previous transformation attempts for *Cuscuta* species, including *C*. *trifolii* [20], *C*. *europaea* [21, 22], and *C*. *reflexa* [23], have yielded only limited success, with varied outcomes in gene integration. Recently, a protocol by Adhikari et al. (2024) [24] outlined a callus-based transformation system for *C*. *campestris*, though the process is lengthy—taking approximately five months—and reported low transformation efficiency. Dependence of that protocol on external plant growth regulators and indirect callus-based regeneration may present limitations, particularly for studies focused on genes associated with plant growth regulation, since *Cuscuta* parasitism relies significantly on these regulators, and Ti plasmid strains affect auxin and cytokinin metabolism [25, 26]. A recent review of Balios and colleagues discuss the various factors affect the growth and development of *Cuscuta* and among them, phytohormones directly regulate haustorium development and functions [27]. Indirect organogenesis could also compromise genetic stability and introduce somaclonal variation, reducing the utility of such systems for comprehensive, high-throughput gene characterization [28].

In light of these challenges, this study aimed to develop an efficient transformation system for *Cuscuta* that would enable high-throughput functional characterization of genes linked to parasitism. Our research sought to establish optimal environmental, nutritional, and procedural conditions to enhance transformation efficiency, focusing on the direct regeneration of transformed tissues while sustaining parasitic growth. This new system offers *Cuscuta* researchers a reliable method for investigating the genetic basis of parasitism, bypassing limitations tied to growth regulators and indirect regeneration, thus enabling more stable, reproducible results.

## Results

### Preparation of explants after seed germination

**Fig 1:**
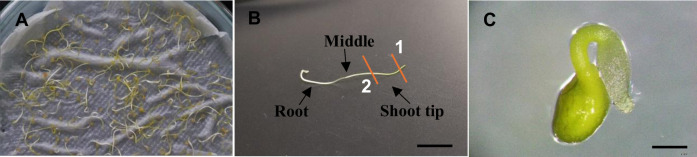
Germinated seedlings used for *in-vitro* culture and transformation. (A) Germinated seedling plate. (B) 3 days old seedling. 1: cut the meristem. 2: cut the tip part. (C) 1-day-old seedling (right after germination). Scale bar B– 1 cm, C– 2 mm.

Sterilized *C*. *campestris* seeds were germinated with 3 cm long shoots after three days of incubation at 29°C in a dark environment (Fig 1A). The shoot tip without the apical meristem, the middle part of the shoot, and the root portion of the shoot (Fig 1B) were used for *in-vitro* growth while the shoot tip without the meristem, the middle part of the shoot of 3-days-old seedling and the root tip of the 1 day-old seedling (Fig 1C) was used for *R*. *rhizogenes*-mediated transformation.

Having a good regeneration protocol from possible explants for inoculation is a key factor for the success of *Rhizobium-*mediated transformation. Since there was no reported tissue culture protocol for *C*. *campestris*, we checked the possibility of direct or indirect regeneration on artificial media. We tested previously optimized media for other species of *Cuscuta in-vitro* culture. Also, we examined two explant factors: age and types of explant.

### Optimization of *in-vitro* growth of *C*. *campestris*

A three-factor experiment was performed to evaluate the regeneration of *C*. *campestris* in different types of explants (shoot tip, middle, root), different culture media (K, MMS), and with different ages of seedlings (3, 5, 7 days old).

**Fig 2:**
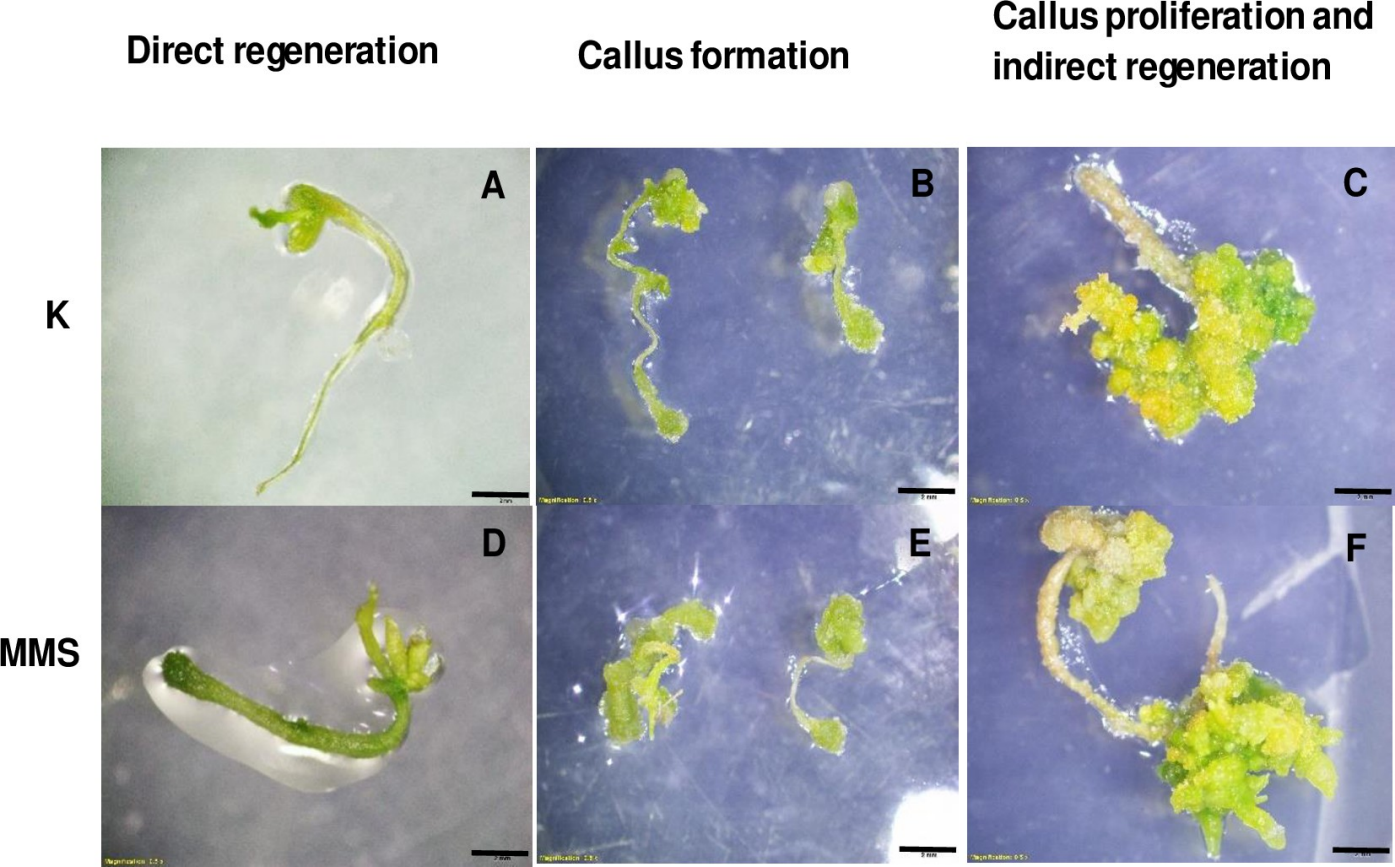
*In-vitro* culture of *C*. *campestris* on artificial media. *In-vitro* growth (A, B, C) on K medium and (D, E, F) on MMS medium. (A, D) Direct regeneration (B, E) callus growth (C) callus proliferation (after 8 weeks) on K medium. (F) Emerging of shoot protuberances—Indirect regeneration (after 8 weeks) on MMS medium. Scale bar A, C, D, F = 2 mm B, E = 5 mm.

Direct regeneration and callus formation commenced 2–3 weeks post-placements on the plates in both K and MMS media (Fig 2A, 2B, 2D and 2E). Over the following weeks, callus proliferation was evident, particularly in K medium where substantial calli were observed after 8 weeks (Fig 2C). Additionally, shoot protuberances emerged as indirect regeneration in MMS medium after the same duration (Fig 2F).

There was a significant interaction among the 3 factors- age, medium, and explant type. Since the interactions had significant effects, the specific effect of each factor on the calli formation and direct regeneration was not evaluated (Table 1). On average, the calli formation or direct regeneration of 7-days-old seedlings was lower in both media compared to 3-days and 5-days-old seedlings suggesting the suitability of young seedlings for *in-vitro* culture. Root explants also showed lower efficiency than shoot tip or middle explants with both media. Middle explants from both 3 and 5-day-old seedlings grown on K medium showed higher calli formation efficiencies of 96% and 64%, respectively. Interestingly, the shoot tip explants of both 3 and 5-day-old seedlings grown on MMS medium showed better direct regeneration efficiencies of 46% and 60% respectively. For raw data, see S1 Table.

**Table 1:** Effect of age of the seedling, culture media, and explant type on calli formation and direct regeneration efficiency of *Cuscuta campestris*.

Age of the seedlings	Culture media	Explant type	Calli formation percentage ± SD	Direct regeneration percentage ± SD
3 days old	K	Shoot tip	54.00±26.07	22.00±25.88
		Middle	96.00±5.47	2±4.47
		Root	36.00±18.16	0.00±0.00
	MMS	Shoot tip	30.00±18.70	46.00±19.49
		Middle	3.33±4.47	0.00±0.00
		Root	2.00±4.47	0.00±0.00
5 days old	K	Shoot tip	40.00±19.23	15.00±26.83
		Middle	64.00±8.94	4.00±5.47
		Root	16.00±13.41	0.00±0.00
	MMS	Shoot tip	10.00±13.03	60.00±29.49
		Middle	6.00±5.47	28.00±16.43
		Root	0.00±0.00	0.00±0.00
7 days old	K	Shoot tip	17.50±15.16	2.50±4.47
		Middle	16.00±25.09	4.00±8.94
		Root	2.00±4.47	0.00±0.00
	MMS	Shoot tip	0.00±0.00	4.00±5.47
		Middle	0.00±0.00	4.00±5.47
		Root	0.00±0.00	0.00±0.00
Age of seedling (A)				
Pr>F			< .0001	0.0001
Culture media (M)				
Pr>F			< .0001	< .0001
Explant type (E)				
Pr>F			< .0001	< .0001
A*M Pr>F			**< .0001**	**0.0033**
A*E Pr>F			**0.0232**	**0.0001**
M*E Pr>F			**< .0001**	**0.0026**
A*M*E Pr>F			**0.0036**	**0.0868**

Each treatment consisted of five plates with 10 explants (each plate is a technical replicate). The efficiency values represent the average ± SD, n = 5 (n = total number of plates per treatment).

Therefore, we selected the shoot tip and middle explants and both K and MMS media for the transformation experiments. Though there was no significance observed between 3-day and 5-day-old seedlings, and because of the demonstrated preference for younger plant tissues in previous transformation experiments [16, 29] we selected 3-day-old seedlings as the choice of explant age for subsequent experiments.

### Optimization of *R*. *rhizogenes-mediated* transformation for *C*. *campestris*

The *R*. *rhizobium*-mediated transformation was done using the streak infection method. First, we tested the explant type, co-incubation period, and culture media on transformation efficiency. To determine the optimal conditions of three explant types; the shoot tip, middle parts of the 3-days-old seedling (Fig 1B), and root tips of the one-day-old seedlings (Fig 1C) were inoculated with two different co-incubation periods one week or two weeks. We used the root tips of 1-day-old seedlings since we utilized *R*. *rhizogenes*, known for its typical use in root transformation. Additionally, three different culture media MS, K, and MMS were tested for each method. We also included the widely used MS [30] medium, as it has previously used for *Cuscuta* transformation [20, 22].

**Fig 3:**
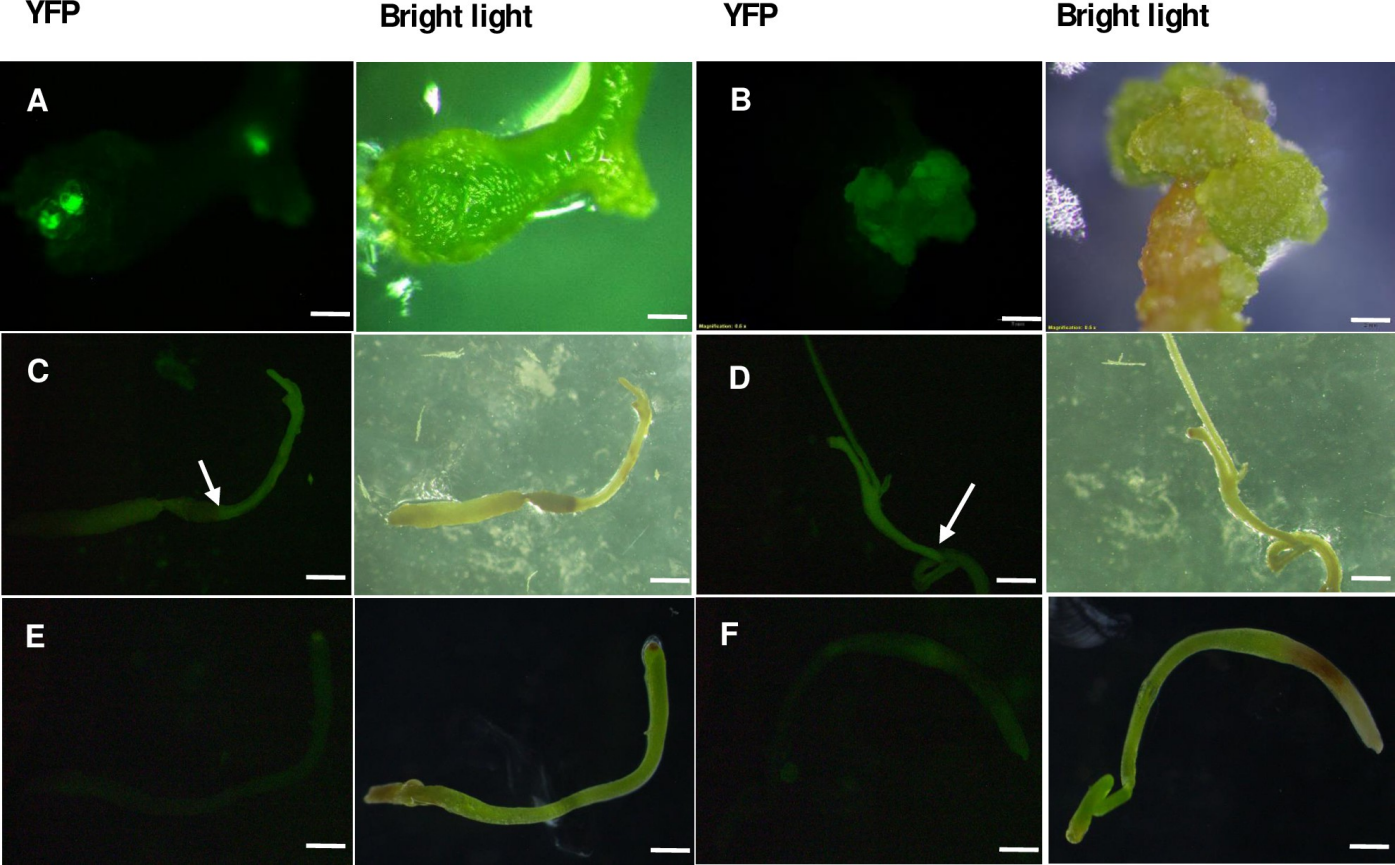
Transformation events reported on artificial media under the fluorescence microscope. (A) YFP expression in *C*. *campestris* cells after 3–4 weeks of the transformation. (B) YFP expressing callus growth. (C-D) YFP expression with regeneration initiation from cut end (Arrow). Initiated shoots did not elongate to make a full plant. (E-F) Explants growing on the same media without *Rhizobium* inoculation did not show any YFP expression. (scale bar A = 0.5 mm, B = 1 mm, C-F = 2 mm).

Transformation events were assessed with YFP expression, under the fluorescence microscope. YFP expression was observed in *C*. *campestris* cells after the 3–4 weeks of transformation (Fig 3A). Transformation efficiency was defined as the percentage of cuttings with YFP expression. Stable transformation efficiency was defined as the percentage of cuttings with YFP expression that showed consistent expression over 6–8 weeks while the transient expression efficiency was defined as the percentage of cuttings that showed temporary YFP expression in transformed tissues. The stable transformation events were observed with MMS and K media, while the basal MS medium did not exhibit stable expression of the YFP (Table 2). However, transient transformation, was evident across all three media, with only the explant type and culture media playing a significant role. For raw data, see S2 Table.

**Table 2:** Effect of culture media, explant type and incubation time in 15°C on YFP expressing stable transformation events and transient transformation of *Cuscuta campestris*.

Culture media	Explant type	Time duration in 15°C	YFP expressing stable transformation events % ± SD	Transient transformation events % ± SD
MS	Shoot tip	1 week	0.00±0.00	6.00±5.47
		2 weeks	0.00±0.00	0.00±0.00
	Middle	1 week	0.00±0.00	0.00±0.00
		2 weeks	0.00±0.00	0.00±0.00
	Root tip	1 week	0.00±0.00	0.00±0.00
		2 weeks	0.00±0.00	0.00±0.00
K	Shoot tip	1 week	10.00±10.00	34.00±34.35
		2 weeks	14.00±19.49	10.00±7.07
	Middle	1 week	0.00±0.00	16.00±23.02
		2 weeks	0.00±0.00	12.00±21.67
	Root tip	1 week	12.00±8.36	0.00±0.00
		2 weeks	12.00±8.36	6.00±8.94
MMS	Shoot tip	1 week	22.00±19.23	4.00±8.94
		2 weeks	14.00±16.73	18.00±21.67
	Middle	1 week	6.00±8.94	30.00±37.41
		2 weeks	0.00±0.00	36.00±46.15
	Root tip	1 week	10.00±10.00	8.00±13.03
		2 weeks	8.00±4.47	4.00±5.47
**Culture media (M)**				
**Pr>F**			0.0001	0.0061
**Explant type (E)**				
**Pr>F**			0.0009	0.0366
Time duration in 15°C (T)				
**Pr>F**			0.4863	0.7425
M*E Pr>F			0.0449	0.0622
M*T Pr>F			0.3246	0.4424
E*T Pr>F			0.9600	0.7836
M*E*T Pr>F			0.9353	0.4197

Each treatment consisted of five plates with 10 explants (each plate is a technical replicate). The efficiency values represent the average ± SD, n = 5 (n = total number of plates per treatment).

Our primary focus was to assess the factors contributing to stable transformation, as the generation of complete transgenic plants relies on the establishment of stable transformed cells. We identified that MMS and K media which contain lower levels of nutrient conditions than normal MS medium play a critical role in the formation of stable transgenic cells.

During the prolonged culture, a YFP-expressing callus was also formed in MMS medium (Fig 3B). Furthermore, stable YFP expression with regeneration initiation from the cut surface was observed in MMS medium (Fig 3C and 3D). The control experiment carried out by dipping the explant in sterilized distilled water did not show any YFP expression (Fig 3E and 3F).

Culture media and explant type collectively contributed to the transformation events significantly while the duration of co-incubation at 15°C or any other interactions did not. Shoot tip explants grown on the MMS medium resulted in the highest stable transformation efficiency of 22% with one-week and 14% with two-week co-incubation periods, which were not statistically significant. According to the results we used shoot tip explants for further experiments.

There were no significant differences in either transient or stable transformation efficiency between the two co-incubation periods, one week or two weeks. As such, an additional one-week incubation period at 15°C did not further increase the transformation rate.

### Optimization of different plant growth regulators for transformation efficiency and initiation

We optimized the suitable parameters to get a better transformation efficiency and elongation of transgenic shoots defined as regeneration. Here we aimed to investigate the impact of different types and concentrations of Auxins and Cytokinins for co-incubation and initiation media on the regeneration of YFP-expressing tissues. In previous experiments, we used MMS medium with 1 mg/L BAP and 3 mg/L NAA for co-incubation. This was named, the regular MMS medium.

We analyzed the data to see the best growth regulator combination for the highest YFP expression in transformation events. When the BAP was added as cytokinin, 5 mg/L of BAP with no NAA in both co-incubation and initiation resulted as the best. When TDZ was cytokinin, the regular MMS medium for co-incubation and 0.05 mg/L TDZ with 0.5 mg/L NAA for initiation worked best (Table 3). We followed up the explants growing on the same media combinations for 6 months with subculturing in 6 weeks and we could see YFP calli and the initiation of YFP regeneration, but we did not see elongation of YFP expressing shoots. For raw data, see S3 Table.

**Table 3:** Transformation efficiency with different hormone combinations for co-incubation and initiation culture.

Media	NAA mg/L	BAP mg/L	TDZ mg/L	Avg % YFP expression ± STD	Remarks
With Co-incubation	0	5		66.67**±**28.28	MMS medium with different Plant growth regulator combinations was used for both co-incubation and the initiation media. Initiation medium consists of 300 mg/L cefotaxime.
		7.5		76.67**±**9.43	
		10		70.00**±**23.57	
		25		55.00**±**21.21	
		50		18.34**±**21.21	
			0.05	55.00**±**21.21	
			0.1	60.00**±**32.99	
			0.2	73.34**±**23.57	
			0.25	66.67**±**23.57	
	0.5	5		56.67**±**18.85	
		7.5		40.00**±**9.43	
		10		35.00**±**11.78	
		25		33.34**±**18.86	
		50		10.00**±**14.14	
			0.05	50.00**±**9.43	
			0.1	43.34**±**9.43	
			0.2	31.67**±**7.07	
			0.25	61.67**±**16.49	
	1	5		38.34**±**2.36	
		7.5		36.67**±**28.28	
		10		53.33**±**23.57	
		25		38.33**±**2.35	
		50		11.67**±**7.07	
			0.05	45.00**±**16.49	
			0.1	51.67**±**25.93	
			0.2	41.67**±**2.36	
			0.25	41.67**±**2.36	
Without Co-incubation	0	5		41.67**±**11.78	For co-incubation, MMS medium with 1 mg/L BAP and 3 mg/L NAA was used. MMS medium with different plant growth regulator combinations were used for initiation medium. Initiation medium consists of 300 mg/L cefotaxime.
		7.5		51.67**±**25.93	
		10		53.34**±**23.57	
		25		45.00**±**16.49	
		50		45.00**±**7.07	
			0.05	50.00**±**23.57	
			0.1	65.00**±**2.36	
			0.2	58.34**±**11.78	
			0.25	51.67**±**11.78	
	0.5	5		70.00**±**9.43	
		7.5		71.67**±**2.36	
		10		80.00**±**14.14	
		25		63.34**±**4.71	
		50		21.67**±**16.49	
			0.05	85.00**±**7.07	
			0.1	76.67**±**18.86	
			0.2	65.00**±**16.49	
			0.25	71.67**±**21.21	
	1	5		61.67**±**30.64	
		7.5		50.00**±**23.57	
		10		60.00**±**37.71	
		25		51.67**±**25.93	
		50		30.00**±**14.14	
			0.05	61.67**±**21.21	
			0.1	45.00**±**21.21	
			0.2	51.67**±**21.21	
			0.25	38.34**±**2.35	
**Composite desirability test**					
If BAP as cytokinin					
Co-incubation medium	0 NAA 5 BAP				
Initiation medium	0 NAA 5 BAP				
If TDZ as cytokinin					
Co-incubation medium	3 NAA 1 BAP (regular MMS)				
Initiation medium	0.5 NAA 0.05 TDZ				

Nevertheless, there was no significant difference between using BAP or TDZ as cytokinin. Therefore, the subsequent experiments focused on the elongation of YFP-expressing shoots, we optimized for a media combination solely with BAP as the cytokinin. This choice was influenced by the cost-effectiveness of BAP compared to TDZ. Additionally, using TDZ for optimal efficiency would require the inclusion of NAA, and our objective was to minimize the use of plant growth regulators in the transformation process. Hence, we chose to proceed with 5 mg/L BAP for growth of transgenic shoots. We observed the tip development initiation in *Cuscuta* cuttings from both cut surfaces. Although 5 mg/L BAP resulted in high transformation efficiency and initiation of transgenic shoots, there was no elongation of these shoot tips.

Furthermore, we examined the orientation of meristem initiation to determine whether the cuttings have the capacity to generate new shoots from both cut surfaces or if there is a directional specificity. This investigation aimed to ascertain whether shoot development is influenced by the concentration of growth regulators and proximity to either the meristem or the root on one side. Interestingly, lower hormone levels prompted tip development on both sides. Our findings suggest that there is no influence from the proximity of the shoot meristem of cuttings while lower hormonal levels may play a key role in inducing new shoot development (Table 4). For raw data, see S4 Table.

**Table 4:** Effect of BAP concentrations on tip growth on the explant from the cut sides.

Treatment	Tip growth on one side	Tip growth on both sides
MMS + 0.5 mg/L NAA + 5 mg/L BAP	22.50 ± 12.58^a^	60.00 ± 14.14^a^
MMS + 0.5 mg/L NAA + 10 mg/L BAP	16.67 ± 5.16^a^	61.67 ± 4.08^a^
MMS + 0.5 mg/L NAA + 25 mg/L BAP	20.00 ± 7.07^a^	42.00 ± 10.95^b^
**Pr > F**	0.5489	0.0139

In a given column values represented by different letters are significantly different (Pr>F = <0.05).

### Involvement of host plant in elongation of transgenic stems

**Fig 4:**
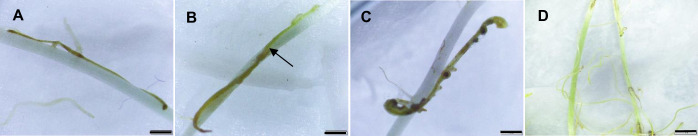
Growth and development of *C*. *campestris* cuttings harvested from outside. (A) Introduction of the cuttings to tomato plants. (B) Development of a new tip from the cut surface (arrow). (C) Development of haustoria. (D) Normal growth on the host was maintained on a plate. (Scale bar A, B, C– 1 mm, D– 1 cm).

Since there was no elongation of transgenic shoots in any of the growth regulators tested, we checked the need for the presence of a host plant and different media for YFP expressing shoot elongation. We used a local tomato variety susceptible to *C*. *campestris*. When we introduced shoot tip parts of *Cuscuta* collected from the wild without meristem directly on the tomato host with no bacterial infection, they developed tips and continued the growth. We checked the difference between cuttings with or without meristems and both of them showed normal elongation with no significant difference. Therefore, we introduced the shoot tip explants from seedlings (used for previous experiments) with bacteria infection to the same susceptible host plant and they also developed a tip and continued the growth (Fig 4A–4D). The growth rate was higher in *Rhizobium*-infected seedling explants than in the wild-collected tips from mature *C*. *campestris* (Table 5). For raw data, see S5 Table.

**Table 5:** Effect of different explant introductions on *C*. *campestris* shoot growth after 10 days on tomato host.

Type of explant compared	Levels	Growth in mm ± SD	Statistics Pr > |t|
Shoot tip parts harvested from wild plants (introduced to the host)	With meristem	7.31 ± 2.33^a^	0.23
	Without meristem	4.99 ± 1.63^a^	
Shoot tip parts from seedlings after bacteria inoculation	With host	15.80 ± 1.20 ^a^	< .0001
	Without host (on media)	0.04 ± 0.03 ^b^	
Shoot tip parts from seedlings and wild plants, without meristem	Seedlings—With bacteria inoculation	15.80 ± 1.20 ^a^	0.0008
	Wild plants—Without bacteria inoculation	4.99 ± 1.63^b^	

Values represented by different letters under each explant compared are significantly different (Pr>F = <0.05).

**Fig 5:**
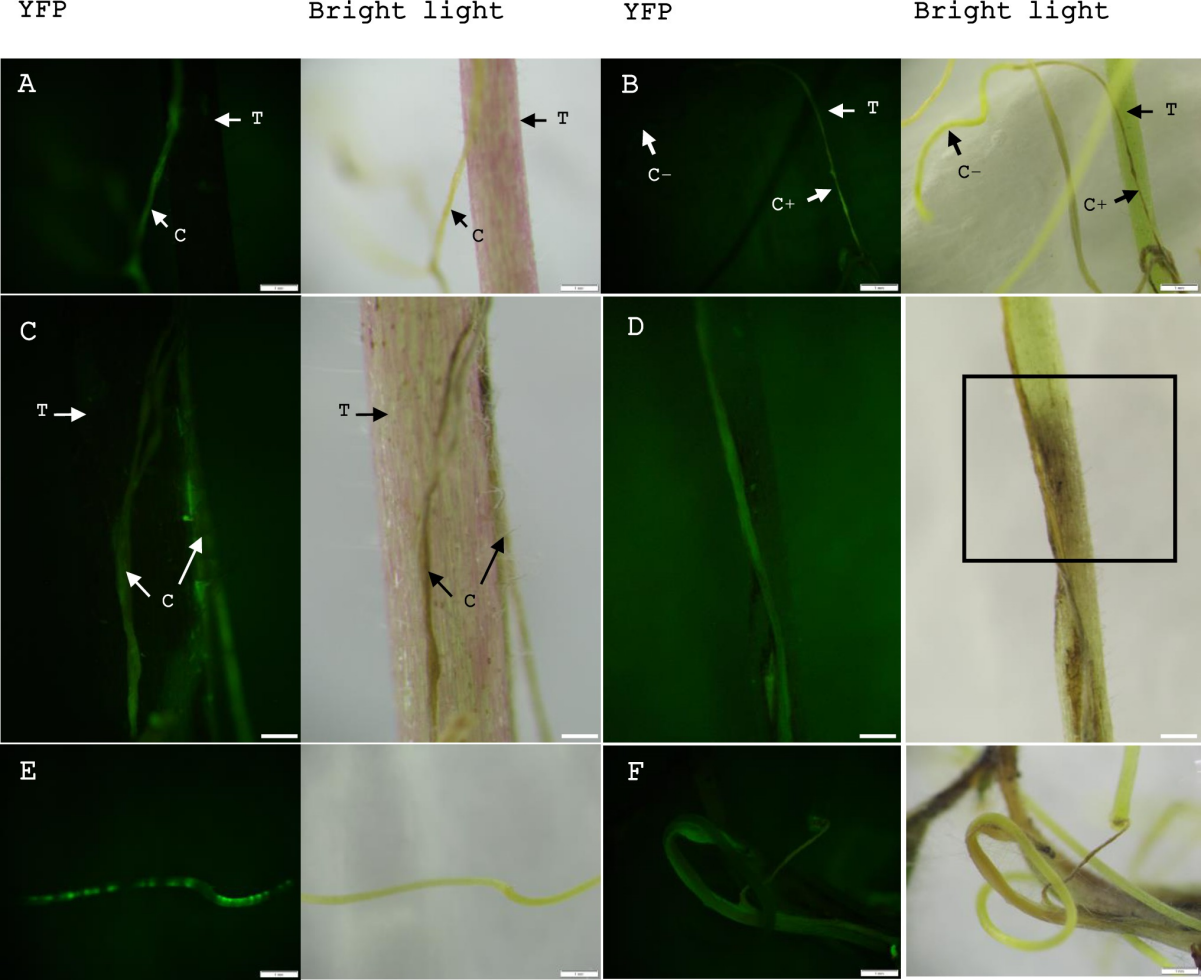
Elongation of transgenic *Cuscuta* shoots expressing YFP that directly transferred to the tomato host after *Rhizobium* inoculation. (A, B, C, D) Elongated YFP expressing *Cuscuta* shoots after one week of transferring to the host. (D) The transgenic *Cuscuta* stem damaged the host. The host stem has turned brown (square). (E) Transient phenotypes and (F) Chimeric phenotypes showing on elongated shoots. C = *Cuscuta*, C+ = YFP positive *Cuscuta*, C- = YFP negative *Cuscuta*, T = Tomato. Scale bar A, B, E, F = 1 mm, C = 0.2 mm, D = 0.5 mm.

We conducted a series of experiments to identify a suitable time of host infection, the effect of co-incubation, and culture conditions. First, we introduced *Cuscuta* explants to the tomato host plant soon after the inoculation of bacteria for co-incubation and subsequent growth (Treatment 1). Many explants made new tips on both sides and continued to grow on tomato plants after one week of co-incubation at 15°C, and about 36% of them showed stable YFP expression. The shoots continued elongation with YFP expression (Fig 5A–5C) and made connections to the host (Fig 5D). About 10.5% showed transient YFP expression (Fig 5E) and few explants showed chimeric YFP expression (Fig 5F).

Furthermore, we explored an alternative medium with different nutrient levels to assess its potential for regenerating YFP-expressing shoots. We included ½ MS medium in our investigations, especially considering that full MS medium initially yielded low transformation efficiency. Concurrently, we optimized hormone levels based on previous tests to enhance the transformation and regeneration process. Therefore, we tested two media, ½ MS and MMS medium with B5 vitamins, and compared them with two different BAP and NAA concentrations and no hormones. For these experiments, we selected the best co-incubation medium among the studied, MMS medium with 5 mg/L of BAP. We initiated three experiments in parallel to test several factors together.

**Fig 6:**
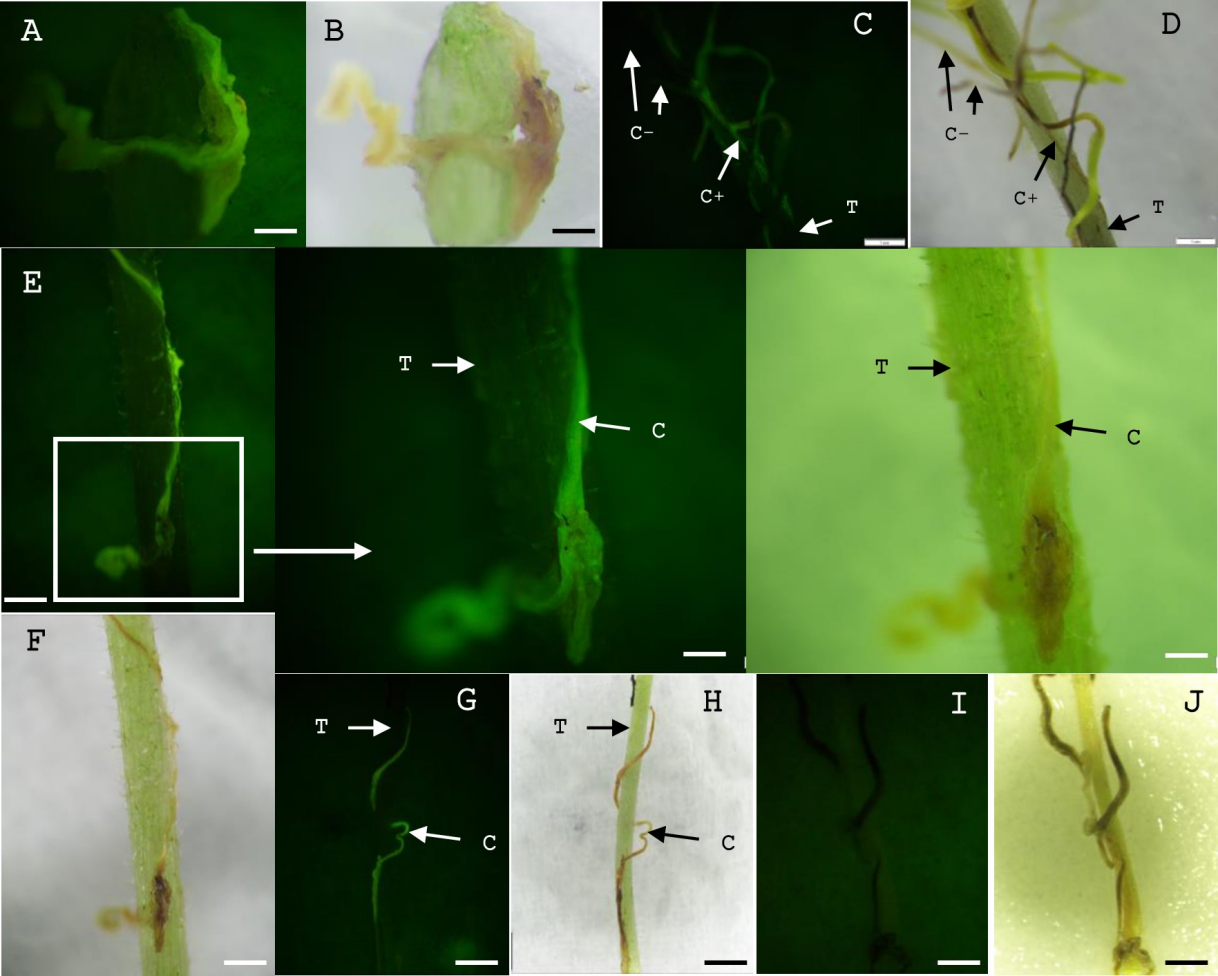
Elongation of transgenic *Cuscuta* stems expressing YFP that co-incubated with *Rhizobium* on MMS media. (A, C, E, G, I, K) images under YFP fluorescence. (B, D, F, H, J, L) images under white light. (A, B, C, D) Elongation of YFP expressing stems which were transferred to the host directly after co-incubation on MMS media and (E, F, G, H) transferred to the host after 5 days on MMS or ½ MS media. (I, J) Explants transferred to the host without *Rhizobium* inoculation. C = *Cuscuta*, C+ = YFP positive *Cuscuta*, C- = YFP negative *Cuscuta*, T = Tomato. Scale bar A, B = 0.2 mm, C, D = 5 mm, E, F = 1 mm (zoomed E, F = 0.2 mm), G, H, I, J = 1 cm.

Another set of explants, co-incubated on MMS medium at 15°C for 10 days, was divided into two groups (Treatment 2). The first group (Set 1), directly introduced to the host plant after co-incubation, exhibited elongation of YFP expressing shoots clearly with tip formation at an efficiency of around 71% (Fig 6A–6D). The other group (Set 2) was transferred to MMS or ½ MS medium with cefotaxime, with or without plant growth regulators, after co-incubation. Those were also introduced to the tomato host after 5 days of culture and resulted in complete transgenic shoots expressing YFP at an efficiency of around 61% (S1 Fig). Regenerated *Cuscuta* shoots displayed stable YFP expression on tomato plants one week after the transfer to the host (Fig 6E–6H). There was no any fluorescence in explants which were transferred to the host without *R*. *rhyzogenes* inoculation (Fig 6I and 6J). Explants that continued growing on the culture media with no host did not elongate YFP expressing shoots during the considered period.

**Fig 7:**
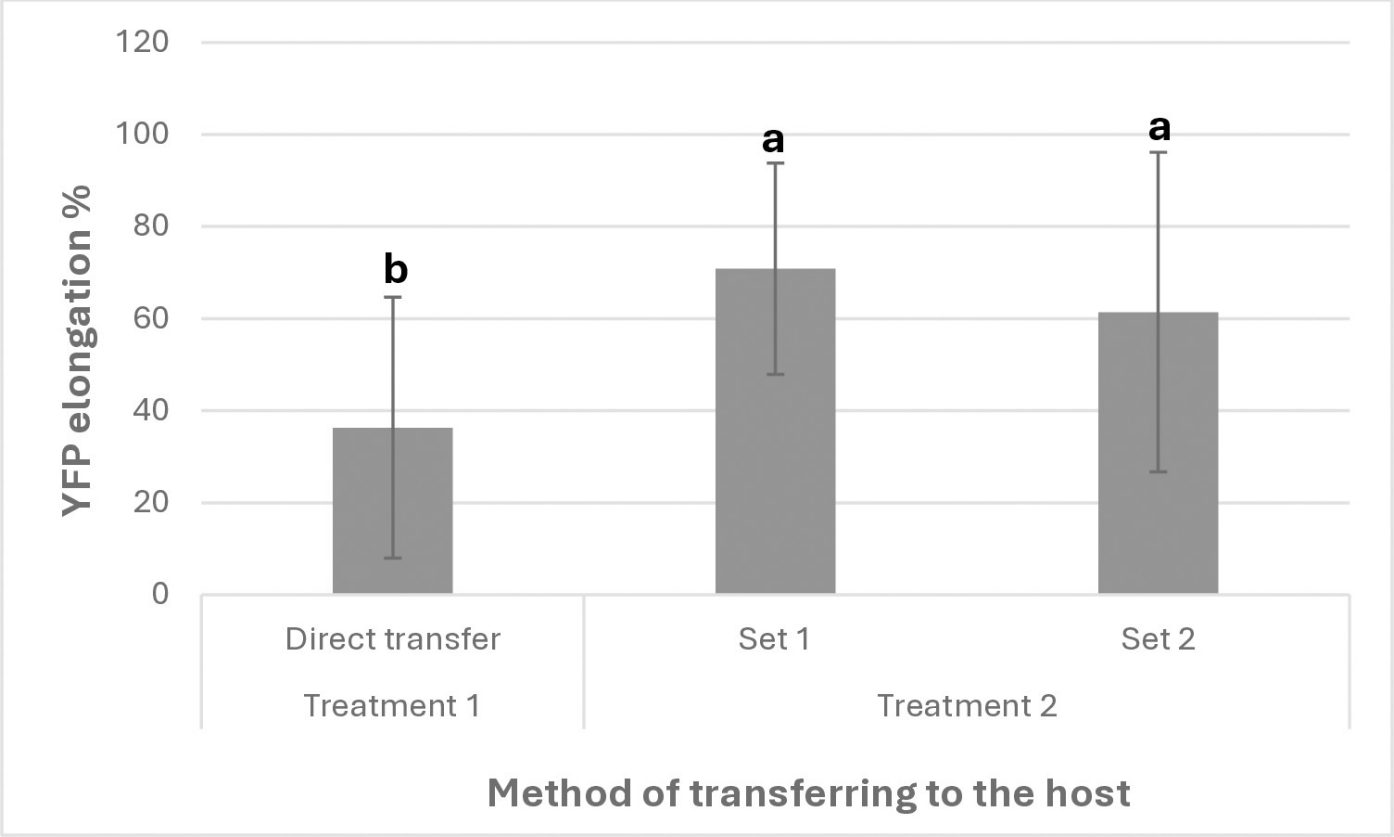
Elongation of YFP expressing shoots transferred to the tomato host at different time points. **Treatment 1.** *Cuscuta* explants were directly transferred to the host after *Rhizobium* inoculation. **Treatment 2.** *Cuscuta* explants were co-incubated with *Rhizobium* on MMS medium. After co-incubation, they were divided into two sets. **Set 1.** Transferred to the host. **Set 2.** Transferred to MMS or ½MS medium and cultured for 5 days and then transferred to the host. Values represented by different letters are significantly different (Pr>F = <0.0001).

As described, one set was transferred to the host after 5 days of incubation on MMS or ½ MS media, with or without plant growth regulators, across four treatments: T1 (0 NAA & 0 BAP), T2 (0 NAA & 10 BAP), T3 (0.5 NAA & 0 BAP), and T4 (0.5 NAA & 10 BAP), with hormone concentrations in mg/L. All explants generated transgenic *Cuscuta* shoots with YFP expression, even in the absence of growth regulators (T1). This indicates that growth regulators are not essential for the elongation of transgenic stems after co-incubation. However, explants transferred to the host after co-incubation with bacteria on MMS medium with 5 mg/L BAP (Fig 7 - Treatment 2 –set 1) exhibited a higher efficiency of YFP expressing shoots, suggesting that co-incubation on MMS medium containing 5 mg/L BAP significantly enhances elongation of transgenic stems expressing YFP efficiently (Fig 7). For raw data, see S6–S8 Tables.

**Fig 8:**
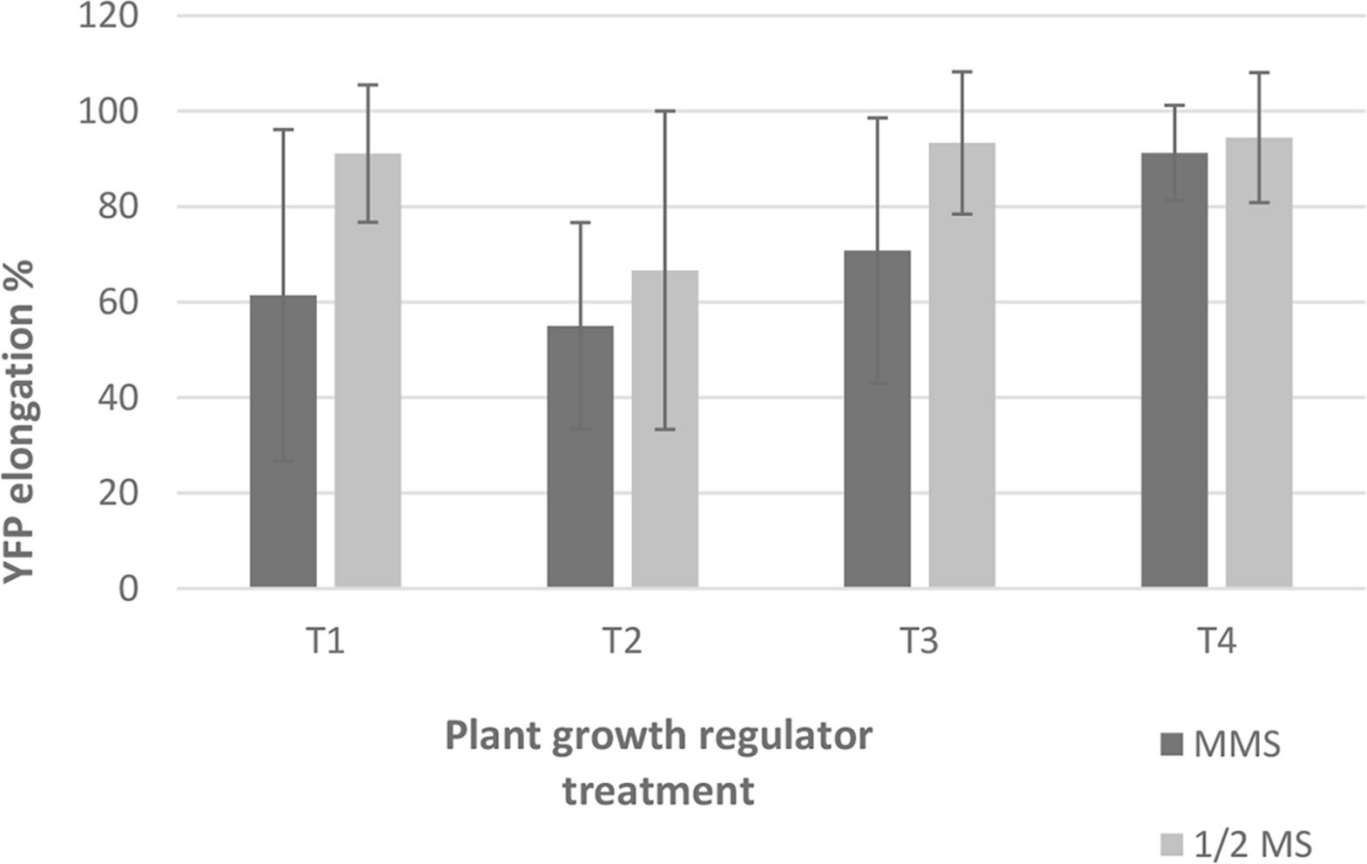
Regeneration efficiency of YFP expressing shoots with different media and plant growth regulator combinations. T1. 0 NAA and 0 BAP, T2. 0 NAA and 10 BAP, T3. 0.5 NAA and 0 BAP, T4. 0.5 NAA and 10 BAP. Each hormone concentration is represented by mg/L. Each value represents mean ± SD of three experiments each with 3 technical replicates.

Culturing the cuttings in different growth regulators and media combinations for five days did not significantly affect the efficiency of elongation of transgenic shoots (Fig 8).

Further, we monitored the growth of randomly selected shoots growing on the host and growing on the media after co-incubation. The explants introduced to the host made a new tip and showed significantly higher growth than the explants which continued the growth on any MMS or ½ MS medium (Table 5).

**Fig 9:**
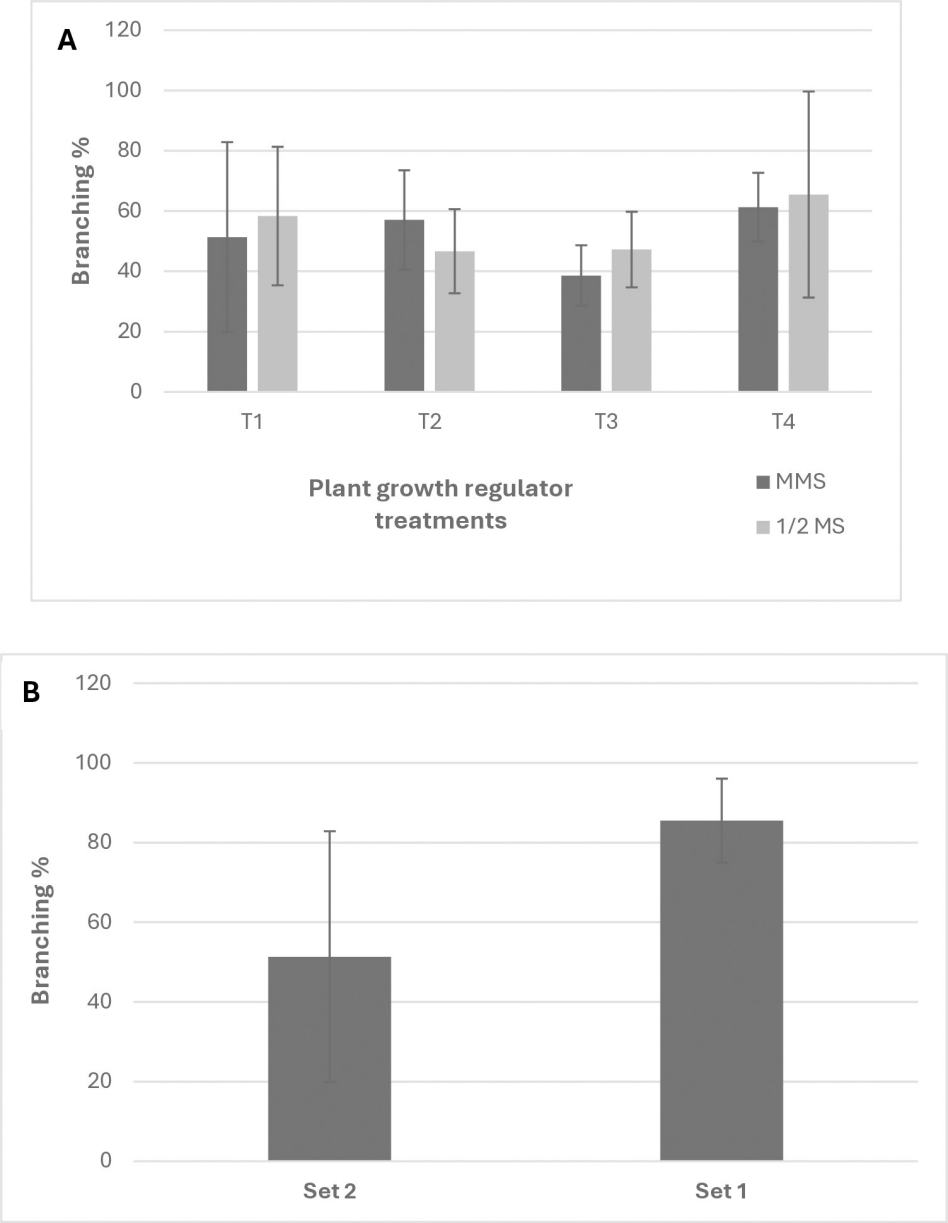
Branching of YFP elongated shoots. (A) Branching with different media and plant growth regulators in treatment 2—set 2. (B) Difference in branching in treatment 2 between set 1 and set 2. (Treatment 2. *Cuscuta* explants were co-incubated with *Rhizobium* on MMS medium. After co-incubation, they were divided into two sets. Set 1. Transferred to the host. Set 2. Transferred to MMS or ½MS medium and cultured for 5 days and then transferred to the host). T1. 0 NAA and 0 BAP, T2. 0 NAA and 10 BAP, T3. 0.5 NAA and 0 BAP, T4. 0.5 NAA and 10 BAP. Each growth regulator concentration is represented by mg/L. Each value represents mean ± SD of three experiments each with 3 technical replicates.

The YFP-expressing elongated shoots made branches about 10 days after being introduced to the susceptible host. About eighty-six (86%) of explants that were introduced to the host after co-incubation developed branches (Set 1). The explants were grown on different media and growth regulators for an additional 5 days before being introduced to the host also developed branches with 51% efficiency (Set 2). There was no significance of media or growth regulators for the branching (Fig 9A). Interestingly, the branching of YFP-expressing shoots was higher in set 1 which was transferred to the host after co-incubation (Fig 9B). For raw data, see S9 and S10 Tables.

Similar results were observed in *in vivo* conditions, where the germinated seedlings were cut and dipped on the lab bench and grown on tomato seedlings germinated under unsterile conditions, maintained at 15°C for 7 days followed by growing in the lab. However, the fungi infection was observed after 10–14 days, though *Cuscuta* continued its active growth (S2 Fig).

It is interesting to note that all the cuttings, including wildtype, stable transgenic, chimeric, and transient, developed multiple haustoria when they were attached to the selected tomato host. However, no haustoria development was observed on cuttings maintained on artificial media. Furthermore, the addition of cefotaxime was not necessary in our system as bacterial growth was not observed after introduction onto the host.

### Confirmation of bacterial gene integration in transgenic *C*. *campestris*

**Fig 10:**
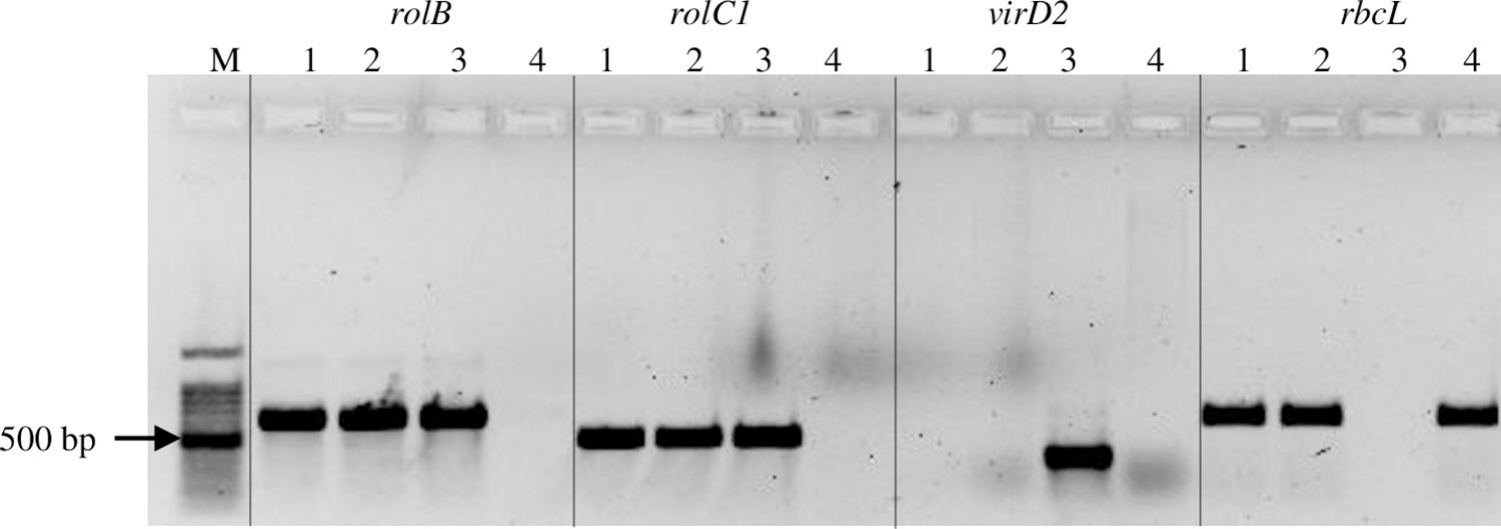
Confirmation of transgene integration via PCR. 1,2: YFP positive *C*. *campestris*, 3: *R*. *rhizogenes*, 4: negative control (non-transformed *C*. *campestris*), M-100 bp molecular weight marker (Promega G210A).

We conducted the standard assessment of the integration of two genes, *rolB* and *rolC1*, as well as *virD2*, through endpoint PCR using cDNA as the template (Fig 10, S3 Fig). We ensured that any contaminating genomic DNA was removed by treating the RNA with DNAase. In YFP-positive *C*. *campestris*, the *rolB* and *rolC1* genes were amplified, while the *virD2* was not present in the same samples. This suggests that the plant genome has successfully incorporated *rolB* and *rolC1*. The *virD2*, which is present on the Ti plasmid and outside of the T-DNA region, does not integrate into the plant genome. It was not amplified in plant samples, but it was amplified in *R*. *rhizogenes*. Non-transformed *C*. *campestris* or the negative control resulted in a product only for the plant endogenous control gene, *rbcL*. Moreover, multiple sequence alignment of *rolB* and *rolC1* genes also revealed the integration of *rolB* and *rolC1* genes with the correct size product in the YFP-positive *C*. *campestris* samples (S4 and S5 Figs).

## Discussion

This study established a highly efficient *Rhizobium rhizogenes*-mediated transformation system for *Cuscuta campestris*, enabling rapid production of transgenic materials suitable for various molecular assays. By optimizing explant selection, media composition, and environmental conditions, our system demonstrated transformation efficiencies of up to 70%, with transgenic shoots ready for analysis within 3–4 weeks. The most effective protocol involved dipping shoot tip explants (with sharp cuts on the tip and proximal end) in a creamy bacterial culture (<24 h old), placing them on MMS medium with 5 mg/L BAP in a 15°C growth chamber with a 16-hour light cycle for 5–10 days, and then transferring them to a host environment at 24°C with 12 hours of light. This study also identified a hormone-free option with 36% efficiency, offering researchers flexibility when studying genes sensitive to growth regulators.

Previous transformation attempts in *Cuscuta* species encountered varied results, with limited gene integration success, lower transformation and subsequent regeneration efficiency, and dependence on external plant growth regulators for a long time, limiting its use in studies investigating genes linked to growth pathways [20–24]. In contrast, our protocol provides a streamlined approach with faster regeneration times and greater efficiency, particularly advantageous for studies where the application of external hormones may interfere with gene functionality related to parasitism, as *Cuscuta* relies heavily on auxin and cytokinin regulation [26, 27, 31].

In developing this transformation protocol, we optimized conditions across multiple variables, such as using fresh, creamy bacterial cultures less than 24 hours old, making precise, sharp cuts on the explants (shoot tip), the flexibility of 15°C co-incubation duration, and nutrient combinations. Investigating explants of different ages and types revealed that tissues at various growth stages, influenced by active metabolism, affect transformation efficiency. These findings align with previous studies considering explant age and orientation [32]. K and MMS media, adapted from other *Cuscuta* species, effectively support callus formation and direct regeneration in *C*. *campestris*. Unlike standard MS medium, these media have reduced macronutrients, and lack Fe.EDTA salts, and include specific growth regulators (S11 Table). This composition shows potential for broader *in vitro* cultivation of *Cuscuta* species. Moreover, the MMS medium with only BAP enhanced the initiation of regeneration of transgenic YFP expression while reducing cost and increasing efficiency compared to TDZ, particularly when minimizing hormonal influence was desired. This protocol was optimized by adjusting these factors, which significantly influenced *C*. *campestris* transformation outcomes. The direct regeneration approach for generating transgenic plants not only reduces the overall cultivation time but also minimizes somaclonal variation and complications associated with plant growth regulators, unlike the indirect regeneration methods.

The host’s presence was crucial for the elongation of *C*. *campestris* transgenic shoots, highlighting the obligate parasitic nature of this plant. In nature, *Cuscuta* seedlings cannot continue growth without host attachment shortly after germination [1]. Host dependence for sustained growth may offer insights into the parasitism mechanisms of *Cuscuta*, particularly regarding hormone signaling pathways necessary for attachment and growth. In our experiments, explants with or without meristems exhibited similar growth when placed on the host, suggesting that host attachment alone is sufficient to support *Cuscuta* growth, thus opening opportunities for deeper exploration of host-plant interactions and potential parasitism-involved genes.

Our first transformation experiment showed that lower nutrient conditions than in MS medium are required for transformation initiation. Incorporating external nutrients or additional plant growth regulators post-co-incubation did not affect the elongation of YFP-expressing shoots, indicating that host-dependent conditions are more relevant for elongation than nutrient availability. Additionally, using sharp cuts, younger seedlings, and maintaining low temperatures were identified as crucial factors in achieving high transformation efficiency, highlighting the specific requirements needed for consistent outcomes in *Cuscuta* transformation. In these experiments, we used YFP as a reporter gene since it has been previously used as an efficient reporter in parasitic plant roots with high amount of secondary metabolites [33, 34]. Nevertheless, the researchers can select any other reporter gene with appropriate microscopic settings and visual reporters such as RUBY, Luciferase, Beta-glucuronidase (GUS), Chloramphenicol Acetyltransferase (CAT), and Antibiotic resistance genes.

Although our system achieves transgenic materials within 6 weeks, we recommend testing it in other *Cuscuta* species, particularly those with economic and ecological importance, to verify its broader applicability. Reports indicate that Cuscuta can establish and make haustoria on non-living surfaces such as metal rods or wooden sticks [35]. Adapting the method for use with different non-living surfaces or other host plants, particularly those less tolerant to low temperatures, may be necessary. The current protocol’s 15°C co-incubation period could be incompatible with cold-sensitive hosts, so modifying the duration may be required. Our recent experiments indicate that even a shortened co-incubation time of 5 days at 15°C did not impact transformation efficiency, suggesting room for further adjustments in host-parasite studies.

Our transformation system, adaptable across *Cuscuta* and potentially other parasitic plants, represents an advancement for functional genomics research. While the hormone-free method offers an effective approach for exploring genes sensitive to growth regulators, more research into other *Cuscuta* species is recommended to fully validate this system. Additionally, expanding studies to include a broader range of host plants will strengthen its applications, especially with cold-sensitive hosts. This transformation system thus provides a valuable tool for advancing parasitic plant research, supporting gene function analysis, and offering insights for agricultural management and ecological conservation through a more profound understanding of plant parasitism.

## Conclusions

This study successfully establishes an efficient *Rhizobium rhizogenes*-mediated transformation system for *Cuscuta campestris*, a significant parasitic plant. By optimizing the explant type, technical procedures, and culture conditions, we achieved stable transformation, rapid initiation of direct regeneration, and continuation of parasitic growth. Using BAP in co-cultivation and initiation media, the transformation efficiency reached 70%. Alternatively, for studies where plant growth regulators may interfere with gene or pathway functions, a hormone-free method achieving 36% efficiency is available. Notably, the discovery of host contact as a key factor for transgenic shoot regeneration provides new insights into plant parasitism mechanisms. This transformation system enables functional characterization of *C*. *campestris* genes and broadens the potential for in vivo transformation and regeneration directly on the host. Overall, this work offers a valuable tool for understanding the biology and genetics of *C*. *campestris* and contributes to the development of effective management strategies for parasitic plants.

## Materials and methods

All the media were prepared in the lab following the standard laboratory procedures and the composition of the media is given in the Supplementary file (S11 Table). While the bacteria medium was solidified with Bacto Agar (Meron), the plant growth medium was solidified with 0.4% Phytagel (Sigma). The cultures were maintained under uniform light conditions at 15°C growth chamber and 24°C culture room; a 16 h light and 8 h dark cycle with a light intensity of 85 μmolm^-2^s^-1^.

The sterilization, micropropagation, and transformation protocols were optimized separately. The yellow fluorescence protein (YFP) expression was tested using an Olympus (SZX10 Japan) Stereo Zoom research florescence microscope equipped with a YFP filter set with excitation HQ490-500, DM505, and emission HQ515-610. The images were captured with a C-mount CCD camera.

### Seed sterilization and germination

*Cuscuta campestris* seeds were collected from an open-pollinated population growing in Peradeniya, Sri Lanka, and the species was confirmed with the National Herbarium of Sri Lanka (PDA). Seeds were surface sterilized using the method previously described by Furuhashi (1991) [36] with some modifications. The seeds were treated with 95% (v/v) H_22_SO_44_ for 1 hour and washed three (3) times with sterile distilled water. Then the seeds were rinsed in 3% (v/v) NaOCl solution, supplemented with Tween-20 for 30 minutes, and washed with sterile distilled water five (5) times. Finally, they were soaked in sterile distilled water at 4°C for 24 hours. Seeds were germinated on wet tissue papers at 29°C in a dark environment (Fig 1A).

### Optimization of *in-vitro* growth of *C*. *campestris*

Two different media were tested; the K medium (modified MS medium containing 1 mg/L Kinetin and 10% coconut water) described by Furuhashi (1991) [36] for *C*. *japonica*, and the MMS-1 medium (modified MS medium containing, 1 mg/L BAP and 3 mg/L NAA, indicated as MMS medium) for *C*. *reflexa* by Srivastava and Dwivedi (2001) [37]. The MMS medium consisted of BAP (6-Benzylaminopurine) instead of BA (6-Benzyladenine) in the current study. We used three different types of explants of about 1 cm in length, shoot tip without meristem, stem region next to the tip (middle), and the root portion of the seedlings (Fig 1B). The age of the seedlings, 3 days, 5 days, and 7 days was considered as another factor. As such, we designed a three-factor factorial experiment to evaluate the best conditions for *C*. *campestris in-vitro* culture.

Each explant was cut sharply and placed horizontally on Petri dishes consisting of appropriate media as 10 cuttings per plate. Each Petri dish 100 mm×15 mm was considered as a technical replicate and each treatment was replicated 5 times. All the cultures were kept in the culture room at 24°C. The number of calli and number of direct regenerated shoots in each replicate were counted after five weeks of initiating cultures and percentage values were calculated accordingly.

### Optimization of *R*. *rhizogenes*-mediated transformation method for *C*. *campestris*

#### Plasmids and bacteria

The R. rhizogenes strain MSU440 contains the plasmid pBIN-YFP, which includes the Aequorea victoria enhanced yellow fluorescent protein (EYFP) gene was kindly provided by the Yoder Lab at UC Davis (33). The EYFP gene is driven by the CaMV 35S promoter for expression in plants [38]. The pBIN-YFP plasmid is 11,775 bp and comprises the kanamycin resistance gene for bacterial selection. Within the T-DNA region, the kanamycin resistance gene is included for plant selection.

*R*. *rhizogenes* was inoculated onto the MGL plates consisting of Kanamycin from glycerol stock and incubated overnight at 29°C to get a single bacterial colony. Then a single colony from the MGL agar plate was inoculated into a 5 mL MGL medium containing Kanamycin and incubated overnight at 29°C while shaking at 200 rpm. Then 1 mL from the 1-day-old bacterial culture was inoculated into an MGL agar plate and incubated overnight at 29°C until creamy bacterial lawn appeared on the plate.

We selected three types of media K, MMS, and MS, three types of explants, shoot tip and middle segments of a 3-day-old seedling, and root of 1 day-old seedling. The streak infection method previously described by Kanchanamala and Bandaranayake (2019) [39] was used for transformation. After making a sharp cut 1 cm from the tip (excluding the meristem) for the shoot tip, the next 1 cm section was taken as the middle part, and all surfaces were carefully dipped into an approximately 18-hour-old *R*. *rhizogenes* culture grown on an MGL plate. Then the cuttings were placed horizontally on the culture plates containing the respective medium.

Controls were performed for all the treatments by dipping the explants into sterilized distilled water. The plates were maintained at 15°C growth chamber for either one week or two weeks for co-incubation. Each Petri dish 100 mm×15 mm consisting of 18–20 explants was considered as a technical replicate and each treatment was replicated at least 5 times.

After the co-incubation period, cuttings were transferred to the same medium supplemented with 300 mg/ L cefotaxime to kill the *R*. *rhizogenes*. Cultures were maintained in a culture room at 24°C.

The number of cuttings with YFP expression per plate was counted after 6 weeks from the date of transformation and percentage values were calculated. In addition, we followed the same tissues for 20 weeks with or without subculturing.

#### Optimization of different plant growth regulators for transformation efficiency and initiation

We examined the MMS medium with 5 different BAP, 4 different TDZ, and 3 different NAA concentrations (Table 3). Shoot tip explants and a week co-incubation period were used.

We followed the cuttings for about 6 months with subculturing on the same media combinations with 300 mg/L cefotaxime while frequently observing under the above microscope for either direct or indirect regeneration.

We defined direct regeneration as growing the YFP expressing shoot without the formation of a callus while indirect regeneration follows a callus stage. The number of cuttings per plate with YFP expressing calli forming new stems and the number of cuttings that directly regenerate new shoots from the cut surface was counted and the percentage values were calculated accordingly.

To investigate the orientation of meristem initiation, we used MMS medium with 0.5 mg/L NAA and three BAP concentrations of 5, 10 and 25 mg/L.

#### Involvement of host plant on elongation of transgenic plants

We used 5–7 days-old tomato seedlings of a local cultivar, Thilina reported to be a susceptible host for *C*. *campestris*. We conducted an experiment assessing the development of 1 cm long *Cuscuta* cuttings collected from the wild on tomato host plants grown on wet hand tissues in Petri dishes. We applied the same methodology for cuttings inoculated with *R*. *rhizogenes* to test whether those elongate in the presence of the host.

**Fig 11:**
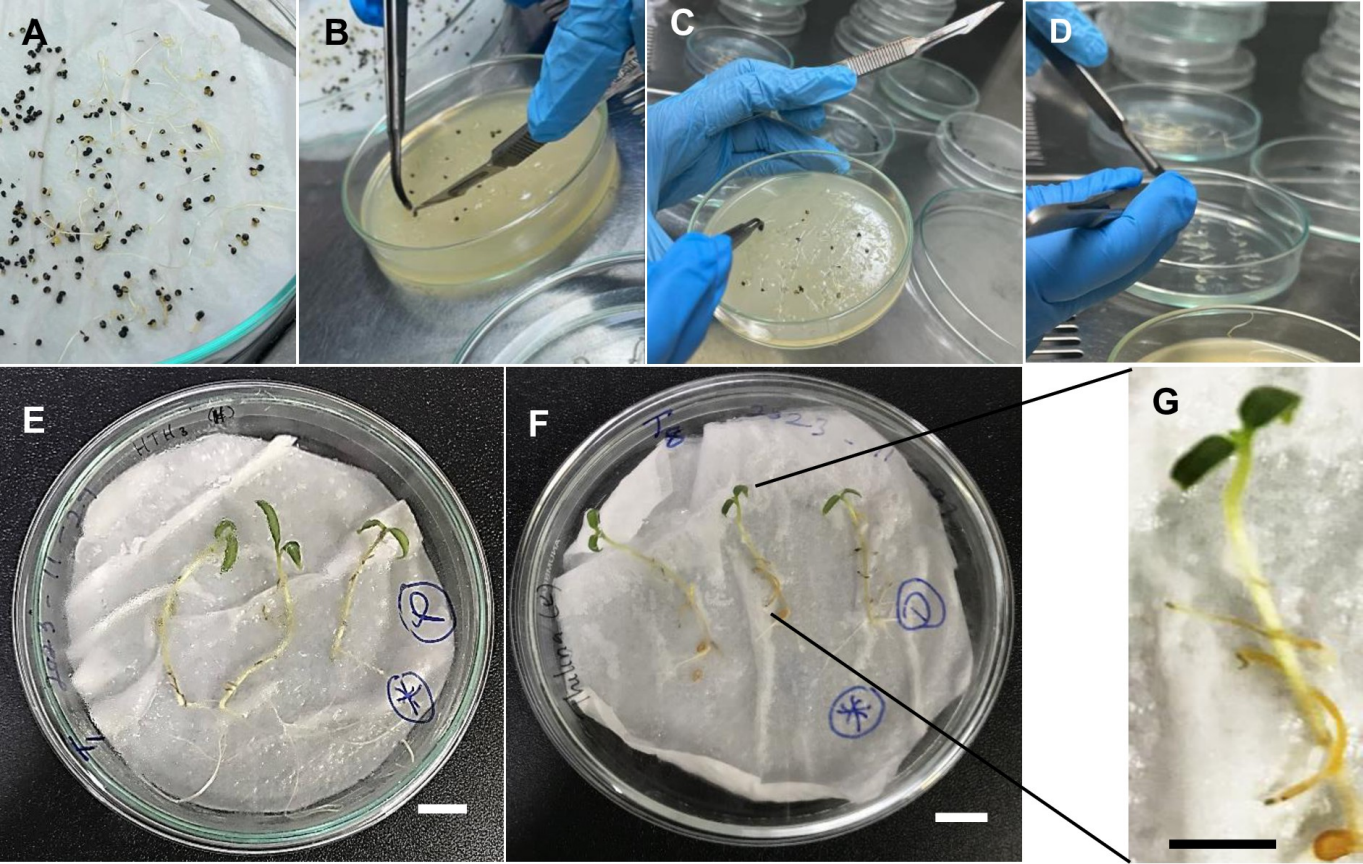
*Rhizobium rhizogenes* mediated transformation procedure for *C*. *campestris*. (A) Three days old germinated seedlings. (B) Cutting the shoot tip. (C) *Rhizobium* inoculation. (D) Placing the cuttings on the respective medium. (E) Cuttings directly placed on host and kept in 15°C for 7–10 days. (F-G) Cuttings transferred onto host after co-incubation on MMS for 10 days and taken picture after 5 days (Scale bar = 1 cm).

Three days old *C*. *campestris* seedlings were used for all these experiments. The shoot tip was cut sharply with a scalpel blade (Fig 11A and 11B). Both surfaces were dipped carefully in about 18 h old *R*. *rhizogenes* culture (Fig 11C) and divided into two treatments, **(01)** placed on host plants facing the tip side towards the shoot tip of the tomato plants grown on sterile hand tissues placed in Petri dishes (Fig 11E) as 3–5 cuttings per plant and **(02)** in Petri dishes with MMS medium with 5 mg/L of BAP as about 10–20 cuttings per plate (Fig 11D). They were transferred to the host after co-incubation on MMS or post co-incubation on MMS or ½ MS media (Fig 11F and 11G). Both treatments were kept in a controlled growth chamber maintained at 15°C for five to ten days.

Plates exposed to treatment 01 were taken out from the 15°C growth chamber after seven days and observed under the YFP fluorescence and the number of cuttings with direct regeneration with stable YFP expression, and the number of cuttings with transient expression, were counted. We also examined chimeric expressions. The transient expression occurs when the genetic materials are taken up by the cell but not incorporated into the cell’s genome [40]. The chimeric expression was defined as the introduction and expression of a gene construct that combines genetic elements from different sources [41]. All the plates were kept in the lab at 24°C and 12 h light and 12 h dark and observed daily under the fluorescence microscope.

The treatment 02 plates taken out from the 15°C growth chamber after 10 days were divided into two sets. The cuttings in the first set were transferred onto tomato plants as same as the treatment 01 and transferred to the lab at 24°C observed under the fluorescence microscope after 7 days and the number of YFP expressing shoots that kept growing was counted. The plates were maintained in the lab at 24°C with 12 h light and 12 h dark.

The cuttings in the second set were transferred to plates containing two different combinations of media (1/2 MS and MMS) and four different concentrations of plant growth regulators (0 NAA and 0 BAP, 0 NAA and 10 BAP, 0.5 NAA and 0 BAP, 0.5 NAA and 10 BAP (all the concentration in mg/L) and 300 mg/L cefotaxime. After 5 days of growth in the culture room at 24°C, 16 h light 8 h dark, the cuttings were transferred separately to tomato plants as in treatment 01. Finally, all the explants from different treatments were transferred to the tomato host for elongation of YFP-expressing transgenic shoots (S1 Fig). The plates were maintained in the lab at 24°C 12 h light and 12 h dark for 7 days and observed under the fluorescence microscope.

Both sets of plants from treatment 02 were further assessed two weeks after introducing them to the host for the ability of the tips to branch. The number of branching YFP tips was counted on each plate.

To measure the growth of transgenic shoots, a set of YFP expressing *Cuscuta* stems was assessed for about 10 days measuring the growth in mm every three days. As a control, young shoot tips harvested from the wild were introduced onto the tomato plants, cutting the tip and without cutting the tip and dipping in water and measuring the growth every three days intervals. Further, the growing transgenic *C*. *campestris* shoots and coiled collected shoots were assessed for their ability to form haustoria, counting the number of YFP-expressing stems forming haustoria 10 days after introducing them to the host.

Further, the above experiments with the hosts were also conducted under unsterile conditions by introducing the *Cuscuta* cuttings onto the host seedlings harvested from the greenhouse and maintained on hand tissues in Petri dishes.

*Cuscuta* cuttings dipped in water were used as the negative control for *R*. *rhizogenes* experiments. As the control for the regeneration experiments of YFP-positive tissues on hosts, YFP-expressing cuttings were maintained on respective media with no host. When we use cuttings, each Petri plate with a minimum of 10 cuttings was considered a replicate and each treatment consisted of a minimum of three plates (Fig 11D). For the plant experiments, each plate with 3–4 tomato plants was considered as a replicate and each experiment was replicated 4–5 times. Each host plant was infected with 3–6 *C*. *campestris* stems (Fig 11E–11G).

#### Confirmation of bacterial gene integration in transgenic *C*. *campestris*

Total RNA was extracted from about five weeks old YFP-positive *C*. *campestris* using the Trizol method following the manufacturer’s instructions (Invitrogen, Cat No 15596–026). The quality and quantity of RNA were determined with a NanoDrop spectrophotometer and Agarose electrophoresis (1%) followed by DNase treatment (DNA -freeTM kit DNase Treatment and Removal, Invitrogen, Cat No:1906). Then cDNA was prepared from 0.5 μg RNA using the Revert Aid First Strand cDNA Synthesis Kit (Thermo Scientific™, Cat No:K1622). Reverse-transcribed RNA was amplified using *rolB*, *rolC1*, *virD2* and *rbcL* genes (Table 6). These genes and the primers have been successfully used in previous work [42–44]. Primers *rolB* and *rolC1* selectively amplify genes present on the T-DNA and incorporate them into the plant genome, while *virD2* is present outside of the T-DNA border and does not integrate into the plant genome and was used as the control to identify the presence of *R*. *rhizogenes* cells. The *rbcL* gene was used as the control for endogenous plant genes.

**Table 6:** Primers used for testing the integration of *R*. *rhizogenes* genes.

Primer	Nucleotide sequence	Expected product size (bp)
***rolB* forward**	5′-CGAGGGGATCCGATTTGCTTT-3′	625
***rolB* reverse**	5′-GACGCCCTCCTCGCCTTCCT-3′	
***rolC1* forward**	5′-TGTGACAAGCAGCGATGAGC-3′	422
***rolC1* reverse**	5′-GATTGCAAACTTGCACTCGC-3′	
***virD2* forward**	5′-ATGCCCGATCGAGCTCAAGT-3′	338
***virD2* reverse**	5´-CCTGACCCAAACATCTCGGCT-3′	
***rbcL* forward**	5´-TGTAAAACGACGGCCAGTATGTCA CCACAAACAGAGACTAAAGC-3′	650
***rbcL* reverse**	5´-CAGGAAACAGCTATGACGTAAAAT CAAGTCCACCRCG-3′	

PCR was carried out in a total of 25 μL containing lX PCR buffer, 1.5 mM MgCl_22_, 0.2 mM dNTP (Promega, USA), 0.2 μM of each primer (Integrated DNA Technologies, Singapore), 2 μL of 1:1 diluted cDNA and 1 Unit Go Taq Flexi DNA polymerase (Promega, USA). The PCR cycle consisted of initial denaturation at 94°C for 3 minutes followed by 35 cycles at 94°C for 30 seconds, 62°C for 30 seconds, 72°C for 1 minute, and a final extension of 72°C for 5 minutes. A well-grown *R*. *rhizogenes* MSU440 colony harboring pBIN-YFP plasmid was suspended in water and used as a positive control. RNA extracted from wild-collected *C*. *campestris* was used as the negative control.

PCR products were separated by electrophoresis (5 Vcm^-1^) on 1.5% agarose gels and stained with ethidium bromide (1 μg/mL). The PCR products of *rolB* and *rolC1* from YFP-positive *C*. *campestris* and *R*. *rhizogenes* were shipped to Macrogen Inc (Seoul, South Korea– http://dna.macrogen.com) for Sanger sequencing using the primers as used for PCR. Chromatograms of the PCR amplified products were visually inspected using Geneious Prime Software (version 11.0.6) for sequencing errors, and the 5’ and 3’ noisy sequences were removed. Then the multiple sequence alignment was carried out for each gene to check the presence of *R*. *rhizogenes* genes in *C*. *campestris*. All the sequences were submitted to GenBank (S12 Table).

### Data analysis

Transformation efficiency was defined as the percentage of cuttings with YFP expression. The percentage values were calculated based on the numbers counted per plate. Each plate was considered as a replicate and all the experiments were replicated at least three times. The anlysis included the *in-vitro* growth efficiency, transformation efficiency, efficiency of elongation of YFP expressing shoots, tip growth and shoot growth, and branching. While the average values of all the replicates ± SD were presented, the total number of plates in treatment was presented as (n). The normality of the data was tested for both growth measurement data and the efficiency data and the data were analyzed with the GLM procedure or the *t-*test using the statistical analysis software SAS OnDemand (Online), SAS Institute Inc. 2024. The mean separation was done using Tukey`s mean separation method.

## Supporting information

S1 Fig The transformation system with different options. (TIFF)

S2 Fig Growth of *Cuscuta* with fungal infections in *in-vivo* conditions (Scale bar = 2 mm). (TIF)

S3 Fig Uncropped gel image for Fig 10. (TIF)

S4 Fig Multiple sequence alignment of *rolB* in YFP positive samples and the *R*. *rhizogenes*. +: *R*. *rhizogenes*, T1 and T2: YFP positive *C*. *campestris*.
(TIF)

S5 Fig Multiple sequence alignment of *rolC1* in YFP positive samples and the *R*. *rhizogenes*. +: *R*. *rhizogenes*, T1 and T2: YFP positive *C*. *campestris*.
(TIF)

S1 Table Raw data for Table 1. (DOCX)

S2 Table Raw data for Table 2. (DOCX)

S3 Table Raw data for Table 3. (DOCX)

S4 Table Raw data for Table 4. (DOCX)

S5 Table Raw data for Table 5. (DOCX)

S6 Table Raw data for Fig 7. (Treatment 1).
(DOCX)

S7 Table Raw data for Fig 7. (Treatment 2 –set 1).
(DOCX)

S8 Table Raw data for Fig 7. (Treatment 2 –set 2).
(DOCX)

S9 Table Raw data for Fig 9. (Treatment 2 –set 2).
(DOCX)

S10 Table Raw data for Fig 9. (Treatment 2 –set 1).
(DOCX)

S11 Table Composition of culture media. (DOCX)

S12 Table GenBank accession numbers. (DOCX)
