# Supplementary figures and images for: An efficient Rhizobium rhizogenes-mediated transformation system for Cuscuta campestris
Source: PLoS One. 2025 Feb 21;20(2):e0317347. doi: 10.1371/journal.pone.0317347 (PMC11844837; doi:10.1371/journal.pone.0317347)

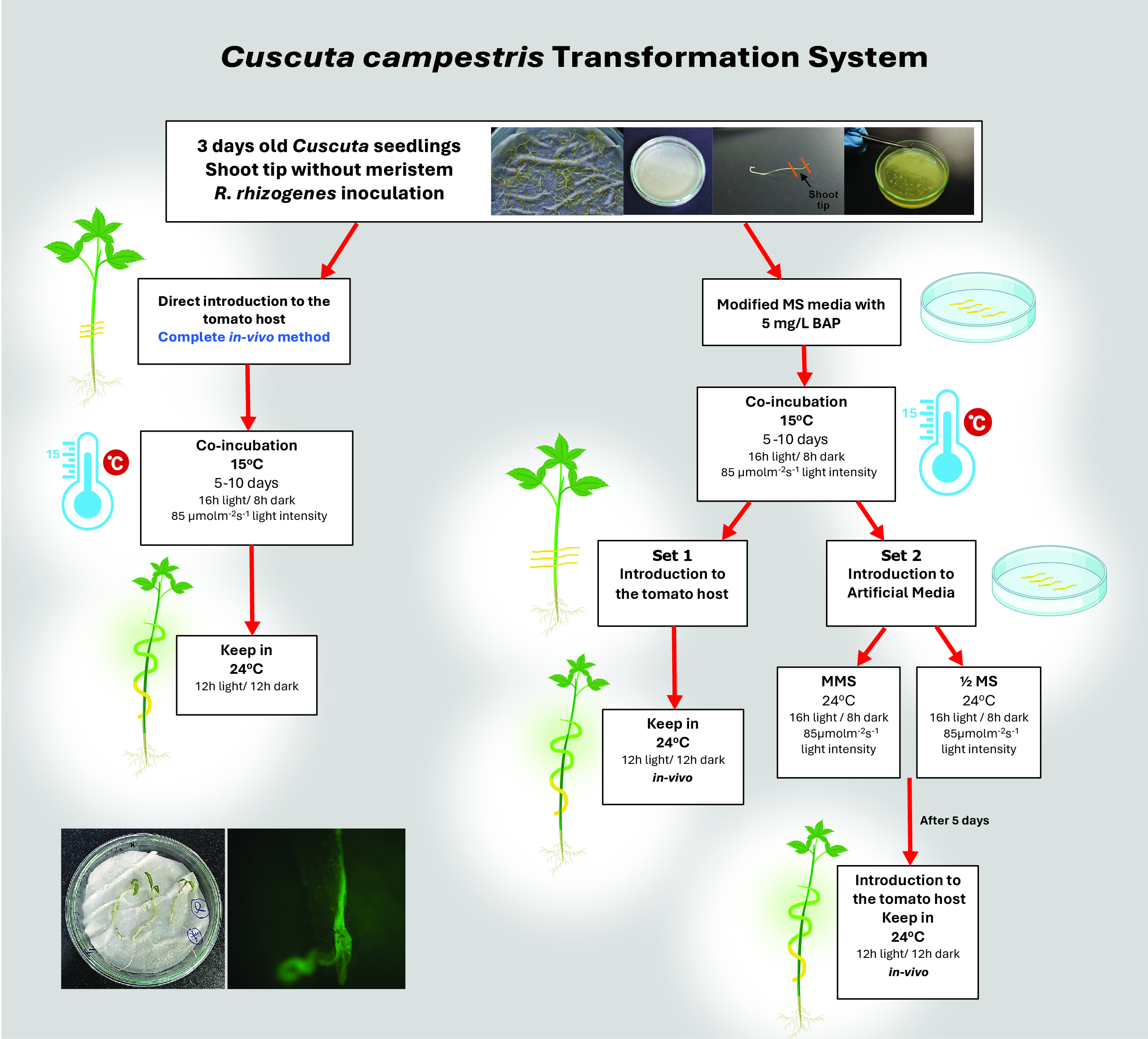

Supplement: S1 Fig — (TIFF) [file pone.0317347.s001.tiff]

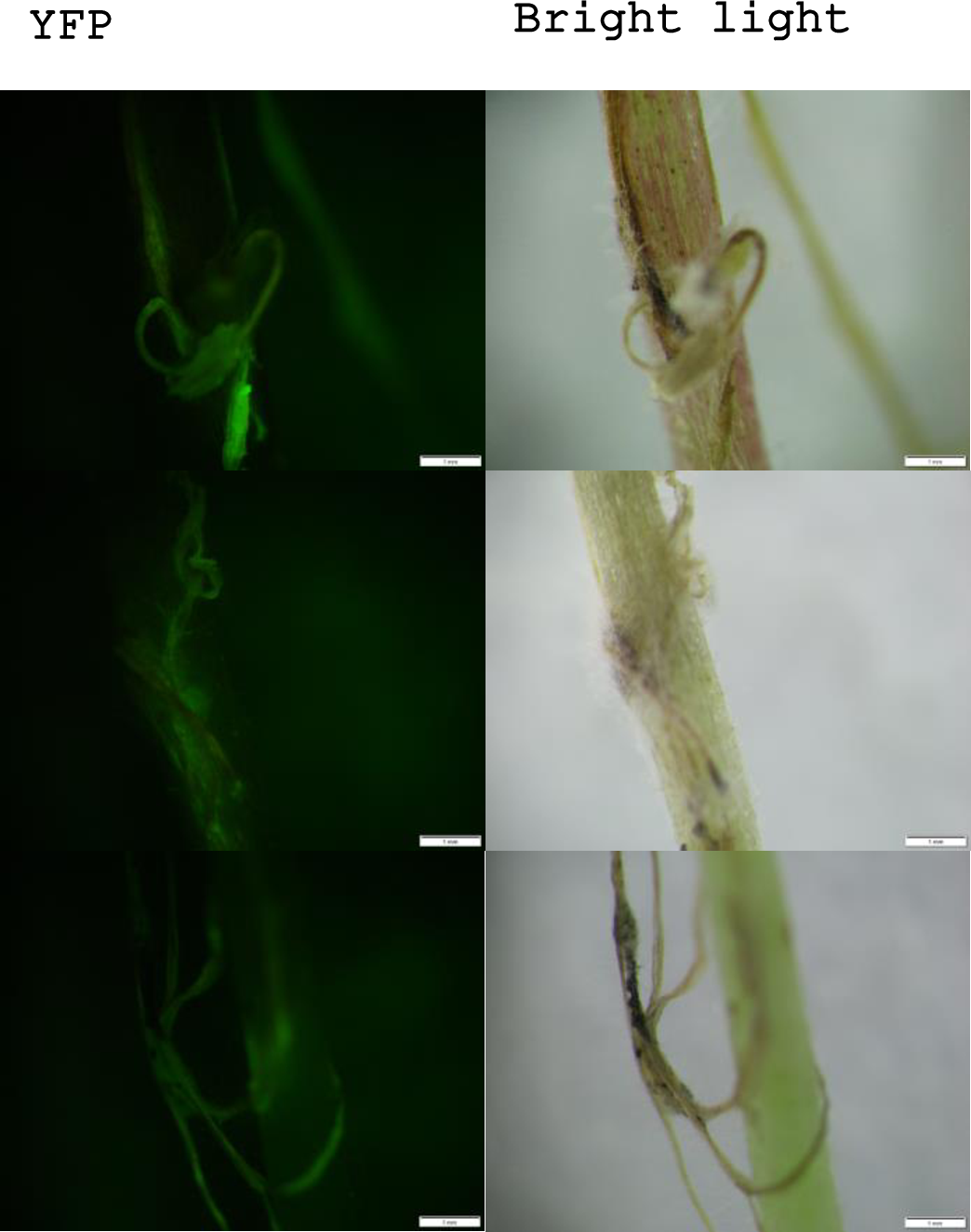

Supplement: S2 Fig — (TIF) [file pone.0317347.s002.tif]

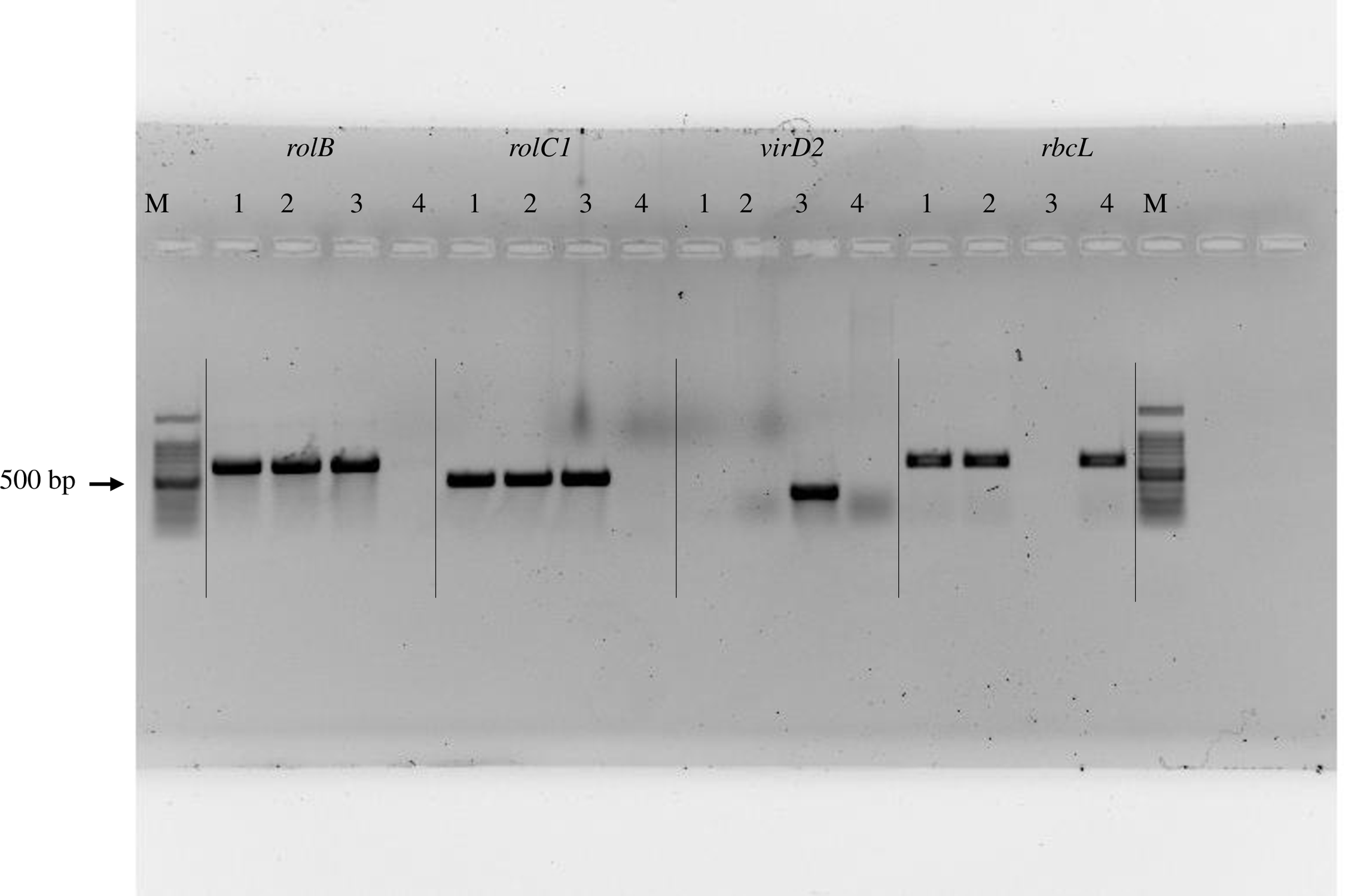

Supplement: S3 Fig — (TIF) [file pone.0317347.s003.tif]

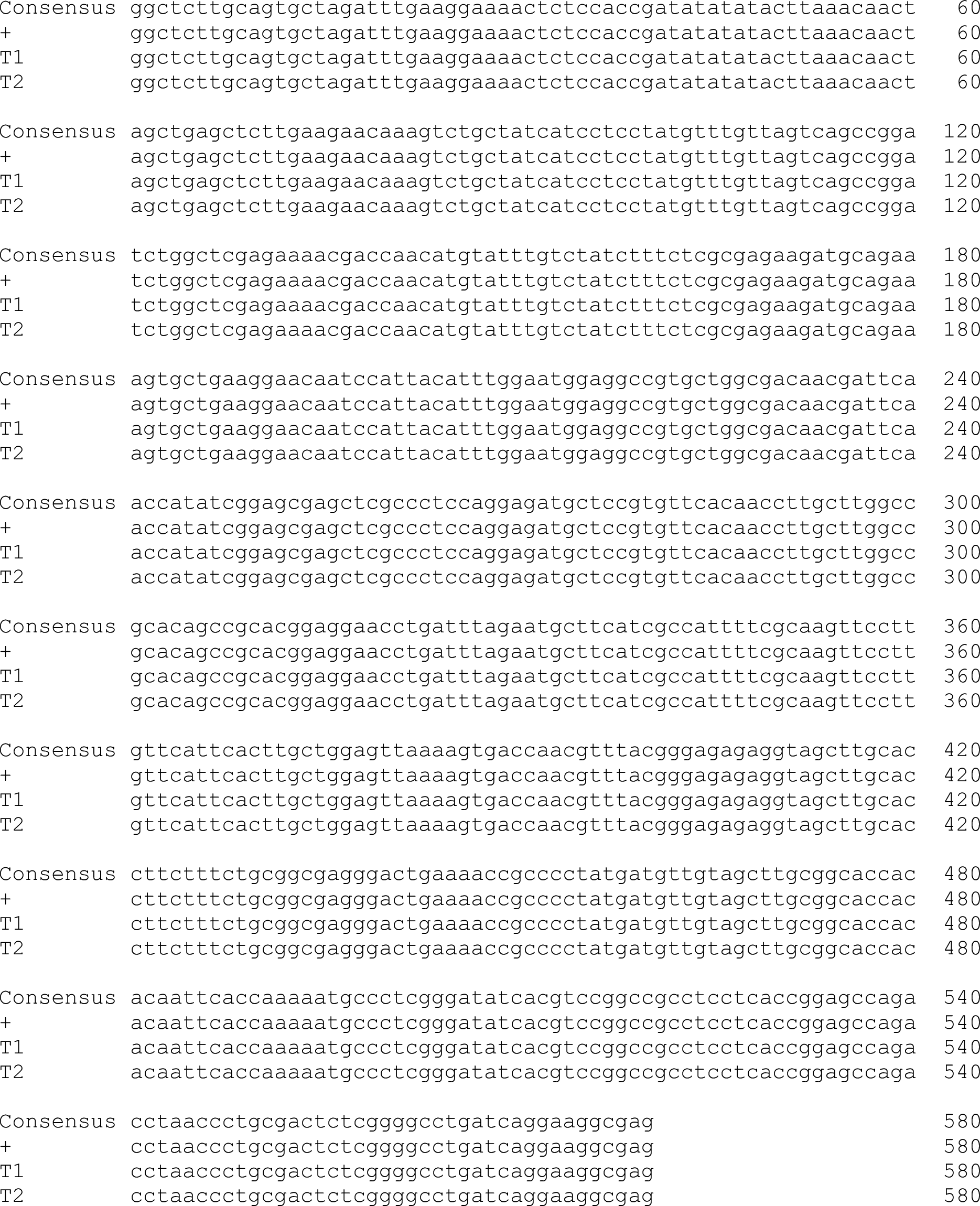

Supplement: S4 Fig — +: R. rhizogenes, T1 and T2: YFP positive C. campestris. (TIF) [file pone.0317347.s004.tif]

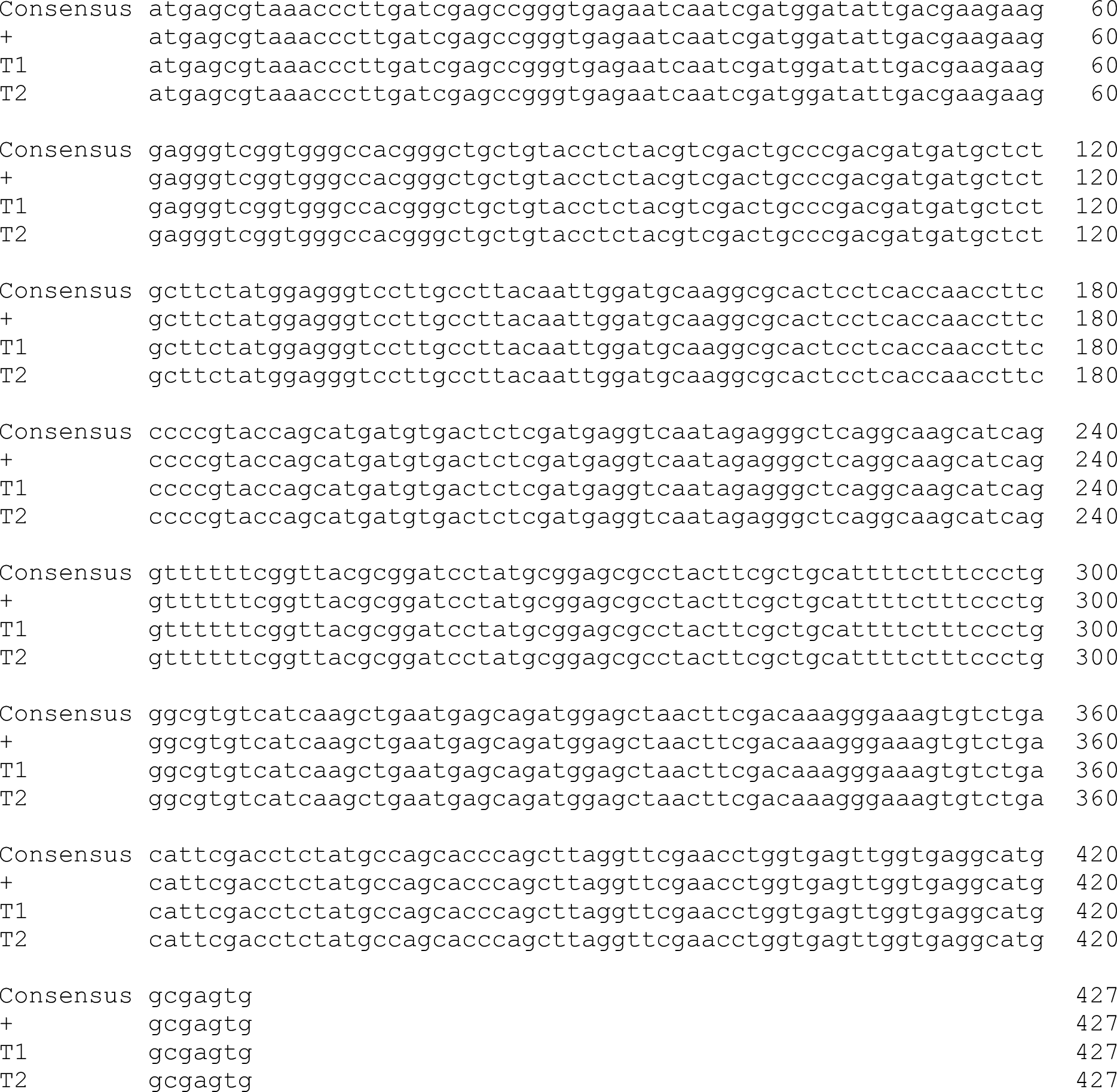

Supplement: S5 Fig — +: R. rhizogenes, T1 and T2: YFP positive C. campestris. (TIF) [file pone.0317347.s005.tif]
