# Supplementary material for: An efficient Rhizobium rhizogenes-mediated transformation system for Cuscuta campestris
Source: PLoS One. 2025 Feb 21;20(2):e0317347. doi: 10.1371/journal.pone.0317347 (PMC11844837; doi:10.1371/journal.pone.0317347)
Supplement: S1 Table — (DOCX) [file pone.0317347.s006.docx]

**S1 Table. Raw data for Table 1**

| **Age of the seedling** | **Medium** | **Explant** | **Plate no.** | **total no of explants** | **No of callus** | **Average callus %** | **No of direct regenerations** | **Average direct regeneration %** |
| --- | --- | --- | --- | --- | --- | --- | --- | --- |
|  |  |  |  |  |  |  |  |  |
| 3 days old | K | apical | i | 10 | 5 | 54 | 5 | 22 |
|  |  |  | ii | 10 | 4 |  | 0 |  |
|  |  |  | iii | 10 | 10 |  | 0 |  |
|  |  |  | iv | 10 | 4 |  | 5 |  |
|  |  |  | v | 10 | 4 |  | 1 |  |
|  |  | middle | i | 10 | 9 | 96 | 1 | 2 |
|  |  |  | ii | 10 | 10 |  | 0 |  |
|  |  |  | iii | 10 | 9 |  | 0 |  |
|  |  |  | iv | 10 | 10 |  | 0 |  |
|  |  |  | v | 10 | 10 |  | 0 |  |
|  |  | root | i | 10 | 1 | 36 | 0 | 0 |
|  |  |  | ii | 10 | 6 |  | 0 |  |
|  |  |  | iii | 10 | 4 |  | 0 |  |
|  |  |  | iv | 10 | 4 |  | 0 |  |
|  |  |  | v | 10 | 3 |  | 0 |  |
|  | MMS 1 | Apical | i | 10 | 1 | 30 | 3 | 46 |
|  |  |  | ii | 10 | 1 |  | 4 |  |
|  |  |  | iii | 10 | 4 |  | 4 |  |
|  |  |  | iv | 10 | 4 |  | 8 |  |
|  |  |  | v | 10 | 5 |  | 4 |  |
|  |  | middle | i | 10 | 1 | 3.33 | 0 | 0 |
|  |  |  | ii | 10 | 0 |  | 0 |  |
|  |  |  | iii | 10 | 0 |  | 0 |  |
|  |  |  | iv | 10 | Contaminated |  |  |  |
|  |  |  | v | 10 | Contaminated |  |  |  |
|  |  | Root | i | 10 | 1 | 2 | 0 | 0 |
|  |  |  | ii | 10 | 0 |  | 0 |  |
|  |  |  | iii | 10 | 0 |  | 0 |  |
|  |  |  | iv | 10 | 0 |  | 0 |  |
|  |  |  | v | 10 | 0 |  | 0 |  |
|  | MS | apical | i | 10 | Contaminated |  |  |  |
|  |  | middle | i | 10 | 0 |  | 0 |  |
|  |  | Root | i | 10 | 0 |  | 0 |  |
| 5 days old | K | apical | i | 10 | 4 | 40 | 6 | 15 |
|  |  |  | ii | 10 | 3 |  | 0 |  |
|  |  |  | iii | 10 | 5 |  | 0 |  |
|  |  |  | iv | 10 | 4 |  | 0 |  |
|  |  |  | v | 10 | Contaminated |  |  |  |
|  |  | middle | i | 10 | 7 | 64 | 1 | 4 |
|  |  |  | ii | 10 | 6 |  | 0 |  |
|  |  |  | iii | 10 | 7 |  | 1 |  |
|  |  |  | iv | 10 | 5 |  | 0 |  |
|  |  |  | v | 10 | 7 |  | 0 |  |
|  |  | root | i | 10 | 3 | 16 | 0 | 0 |
|  |  |  | ii | 10 | 0 |  | 0 |  |
|  |  |  | iii | 10 | 3 |  | 0 |  |
|  |  |  | iv | 10 | 1 |  | 0 |  |
|  |  |  | v | 10 | 1 |  | 0 |  |
|  | MMS 1 | apical | i | 10 | 3 | 10 | 6 | 60 |
|  |  |  | ii | 10 | 1 |  | 5 |  |
|  |  |  | iii | 10 | 0 |  | 5 |  |
|  |  |  | iv | 10 | 0 |  | 8 |  |
|  |  |  | v | 10 | Contaminated |  |  |  |
|  |  | middle | i | 10 | 1 | 6 | 4 | 28 |
|  |  |  | ii | 10 | 0 |  | 2 |  |
|  |  |  | iii | 10 | 0 |  | 2 |  |
|  |  |  | iv | 10 | 1 |  | 5 |  |
|  |  |  | v | 10 | 1 |  | 1 |  |
|  |  | root | i | 10 | Contaminated | 0 |  | 0 |
|  |  |  | ii | 10 | 0 |  | 0 |  |
|  |  |  | iii | 10 | 0 |  | 0 |  |
|  |  |  | iv | 10 | 0 |  | 0 |  |
|  |  |  | v | 10 | 0 |  | 0 |  |
|  | MS | apical | i | 10 | 0 |  | 0 |  |
|  |  | middle | i | 10 | 0 |  | 0 |  |
|  |  | root | i | 10 | 0 |  | 0 |  |
| 7 days old | K | apical | i | 10 | 1 | 17.5 | 0 | 2.5 |
|  |  |  | ii | 10 | 3 |  | 0 |  |
|  |  |  | iii | 10 | 3 |  | 0 |  |
|  |  |  | iv | 10 | 0 |  | 1 |  |
|  |  |  | v | 10 | contaminated |  |  |  |
|  |  | middle | i | 10 | 0 | 16 | 2 | 4 |
|  |  |  | ii | 10 | 6 |  | 0 |  |
|  |  |  | iii | 10 | 1 |  | 0 |  |
|  |  |  | iv | 10 | 0 |  | 0 |  |
|  |  |  | v | 10 | 1 |  | 0 |  |
|  |  | root | i | 10 | 0 | 2 | 0 | 0 |
|  |  |  | ii | 10 | 0 |  | 0 |  |
|  |  |  | iii | 10 | 0 |  | 0 |  |
|  |  |  | iv | 10 | 0 |  | 0 |  |
|  |  |  | v | 10 | 1 |  | 0 |  |
|  | MMS 1 | apical | i | 10 | 0 | 0 | 0 | 4 |
|  |  |  | ii | 10 | 0 |  | 1 |  |
|  |  |  | iii | 10 | 0 |  | 1 |  |
|  |  |  | iv | 10 | 0 |  | 0 |  |
|  |  |  | v | 10 | 0 |  | 0 |  |
|  |  | middle | i | 10 | 0 | 0 | 0 | 4 |
|  |  |  | ii | 10 | 0 |  | 1 |  |
|  |  |  | iii | 10 | 0 |  | 0 |  |
|  |  |  | iv | 10 | 0 |  | 1 |  |
|  |  |  | v | 10 | 0 |  | 0 |  |
|  |  | root | i | 10 | 0 | 0 | 0 | 0 |
|  |  |  | ii | 10 | 0 |  | 0 |  |
|  |  |  | iii | 10 | 0 |  | 0 |  |
|  |  |  | iv | 10 | 0 |  | 0 |  |
|  |  |  | v | 10 | contaminated |  |  |  |
