# Supplementary material for: An efficient Rhizobium rhizogenes-mediated transformation system for Cuscuta campestris
Source: PLoS One. 2025 Feb 21;20(2):e0317347. doi: 10.1371/journal.pone.0317347 (PMC11844837; doi:10.1371/journal.pone.0317347)
Supplement: S2 Table — (DOCX) [file pone.0317347.s007.docx]

**S2 Table. Raw data for Table 2**

| **Culture medium** | **Explant** | **Time duration in 15^o^C** | **Plate no.** | **Total no of explants** | **No of YFP stable events** | **YFP stable events %** | **No of YFP transient events** | **YFP transient events %** |
| --- | --- | --- | --- | --- | --- | --- | --- | --- |
| MS | Shoot tip | 1 | i | 10 | 0 | 0 | 1 | 6 |
|  |  |  | ii | 10 | 0 |  | 0 |  |
|  |  |  | iii | 10 | 0 |  | 0 |  |
|  |  |  | iv | 10 | 0 |  | 1 |  |
|  |  |  | v | 10 | 0 |  | 1 |  |
|  |  | 2 | i | 10 | 0 | 0 | 0 | 0 |
|  |  |  | ii | 10 | 0 |  | 0 |  |
|  |  |  | iii | 10 | 0 |  | 0 |  |
|  |  |  | iv | 10 | 0 |  | 0 |  |
|  |  |  | v | 10 | 0 |  | 0 |  |
|  | Middle | 1 | i | 10 | 0 | 0 | 0 | 0 |
|  |  |  | ii | 10 | 0 |  | 0 |  |
|  |  |  | iii | 10 | 0 |  | 0 |  |
|  |  |  | iv | 10 | 0 |  | 0 |  |
|  |  |  | v | 10 | 0 |  | 0 |  |
|  |  | 2 | i | 10 | 0 | 0 | 0 | 0 |
|  |  |  | ii | 10 | 0 |  | 0 |  |
|  |  |  | iii | 10 | 0 |  | 0 |  |
|  |  |  | iv | 10 | 0 |  | 0 |  |
|  |  |  | v | 10 | 0 |  | 0 |  |
|  | Root tip | 1 | i | 10 | 0 | 0 | 0 | 0 |
|  |  |  | ii | 10 | 0 |  | 0 |  |
|  |  |  | iii | 10 | 0 |  | 0 |  |
|  |  |  | iv | 10 | 0 |  | 0 |  |
|  |  |  | v | 10 | 0 |  | 0 |  |
|  |  | 2 | i | 10 | 0 | 0 | 0 | 0 |
|  |  |  | ii | 10 | 0 |  | 0 |  |
|  |  |  | iii | 10 | 0 |  | 0 |  |
|  |  |  | iv | 10 | 0 |  | 0 |  |
|  |  |  | v | 10 | 0 |  | 0 |  |
| K | Shoot tip | 1 | i | 10 | 2 | 10 | 6 | 34 |
|  |  |  | ii | 10 | 0 |  | 0 |  |
|  |  |  | iii | 10 | 0 |  | 1 |  |
|  |  |  | iv | 10 | 1 |  | 2 |  |
|  |  |  | v | 10 | 2 |  | 8 |  |
|  |  | 2 | i | 10 | 0 | 14 | 2 | 10 |
|  |  |  | ii | 10 | 3 |  | 1 |  |
|  |  |  | iii | 10 | 0 |  | 0 |  |
|  |  |  | iv | 10 | 0 |  | 1 |  |
|  |  |  | v | 10 | 4 |  | 1 |  |
|  | Middle | 1 | i | 10 | 0 | 0 | 0 | 16 |
|  |  |  | ii | 10 | 0 |  | 0 |  |
|  |  |  | iii | 10 | 0 |  | 0 |  |
|  |  |  | iv | 10 | 0 |  | 5 |  |
|  |  |  | v | 10 | 0 |  | 3 |  |
|  |  | 2 | i | 10 | 0 | 0 | 0 | 12 |
|  |  |  | ii | 10 | 0 |  | 0 |  |
|  |  |  | iii | 10 | 0 |  | 5 |  |
|  |  |  | iv | 10 | 0 |  | 1 |  |
|  |  |  | v | 10 | 0 |  | 0 |  |
|  | Root tip | 1 | i | 10 | 0 | 12 | 0 | 0 |
|  |  |  | ii | 10 | 1 |  | 0 |  |
|  |  |  | iii | 10 | 1 |  | 0 |  |
|  |  |  | iv | 10 | 2 |  | 0 |  |
|  |  |  | v | 10 | 2 |  | 0 |  |
|  |  | 2 | i | 10 | 2 | 12 | 2 | 6 |
|  |  |  | ii | 10 | 2 |  | 1 |  |
|  |  |  | iii | 10 | 1 |  | 0 |  |
|  |  |  | iv | 10 | 0 |  | 0 |  |
|  |  |  | v | 10 | 1 |  | 0 |  |
| MMS | Shoot tip | 1 | i | 10 | 2 | 22 | 0 | 4 |
|  |  |  | ii | 10 | 1 |  | 0 |  |
|  |  |  | iii | 10 | 0 |  | 0 |  |
|  |  |  | iv | 10 | 5 |  | 0 |  |
|  |  |  | v | 10 | 3 |  | 2 |  |
|  |  | 2 | i | 10 | 1 | 14 | 0 | 18 |
|  |  |  | ii | 10 | 0 |  | 0 |  |
|  |  |  | iii | 10 | 0 |  | 1 |  |
|  |  |  | iv | 10 | 2 |  | 5 |  |
|  |  |  | v | 10 | 4 |  | 3 |  |
|  | Middle | 1 | i | 10 | 0 | 6 | 0 | 30 |
|  |  |  | ii | 10 | 2 |  | 1 |  |
|  |  |  | iii | 10 | 0 |  | 0 |  |
|  |  |  | iv | 10 | 1 |  | 8 |  |
|  |  |  | v | 10 | 0 |  | 6 |  |
|  |  | 2 | i | 10 | 0 | 0 | 1 | 36 |
|  |  |  | ii | 10 | 0 |  | 0 |  |
|  |  |  | iii | 10 | 0 |  | 10 |  |
|  |  |  | iv | 10 | 0 |  | 7 |  |
|  |  |  | v | 10 | 0 |  | 0 |  |
|  | Root tip | 1 | i | 10 | 2 | 10 | 3 | 8 |
|  |  |  | ii | 10 | 0 |  | 0 |  |
|  |  |  | iii | 10 | 1 |  | 0 |  |
|  |  |  | iv | 10 | 2 |  | 0 |  |
|  |  |  | v | 10 | 0 |  | 1 |  |
|  |  | 2 | i | 10 | 1 | 8 | 1 | 4 |
|  |  |  | ii | 10 | 1 |  | 0 |  |
|  |  |  | iii | 10 | 1 |  | 0 |  |
|  |  |  | iv | 10 | 0 |  | 1 |  |
|  |  |  | v | 10 | 1 |  | 0 |  |
