# Supplementary material for: An efficient Rhizobium rhizogenes-mediated transformation system for Cuscuta campestris
Source: PLoS One. 2025 Feb 21;20(2):e0317347. doi: 10.1371/journal.pone.0317347 (PMC11844837; doi:10.1371/journal.pone.0317347)
Supplement: S3 Table — (DOCX) [file pone.0317347.s008.docx]

**S3 Table. Raw data for Table 3**

|  | **NAA**  **mg/L** | **BAP**  **mg/L** | **TDZ**  **mg/L** | **No of explants per plate** | **No of YFP stable events Plate 1** | **YFP stable events % Plate 1** | **No of YFP stable events Plate 2** | **YFP stable events % Plate 2** | **Average YFP stable events %** |
| --- | --- | --- | --- | --- | --- | --- | --- | --- | --- |
| With Co-incubation | 0 | 5 |  | 30 | 26 | 86.67 | 14 | 46.67 | 66.67 |
|  |  | 7.5 |  | 30 | 21 | 70 | 25 | 83.33 | 76.67 |
|  |  | 10 |  | 30 | 16 | 53.33333333 | 26 | 86.6666667 | 70.00 |
|  |  | 25 |  | 30 | 21 | 70 | 12 | 40 | 55.00 |
|  |  | 50 |  | 30 | 1 | 3.333333333 | 10 | 33.3333333 | 18.33 |
|  |  |  | 0.05 | 30 | 21 | 70 | 12 | 40 | 55.00 |
|  |  |  | 0.1 | 30 | 25 | 83.33333333 | 11 | 36.6666667 | 60.00 |
|  |  |  | 0.2 | 30 | 27 | 90 | 17 | 56.6666667 | 73.33 |
|  |  |  | 0.25 | 30 | 25 | 83.33333333 | 15 | 50 | 66.67 |
|  | 0.5 | 5 |  | 30 | 21 | 70 | 13 | 43.3333333 | 56.67 |
|  |  | 7.5 |  | 30 | 14 | 46.66666667 | 10 | 33.3333333 | 40.00 |
|  |  | 10 |  | 30 | 13 | 43.33333333 | 8 | 26.6666667 | 35.00 |
|  |  | 25 |  | 30 | 14 | 46.66666667 | 6 | 20 | 33.33 |
|  |  | 50 |  | 30 | 6 | 20 | 0 | 0 | 10.00 |
|  |  |  | 0.05 | 30 | 17 | 56.66666667 | 13 | 43.3333333 | 50.00 |
|  |  |  | 0.1 | 30 | 15 | 50 | 11 | 36.6666667 | 43.33 |
|  |  |  | 0.2 | 30 | 11 | 36.66666667 | 8 | 26.6666667 | 31.67 |
|  |  |  | 0.25 | 30 | 22 | 73.33333333 | 15 | 50 | 61.67 |
|  | 1 | 5 |  | 30 | 12 | 40 | 11 | 36.6666667 | 38.33 |
|  |  | 7.5 |  | 30 | 17 | 56.66666667 | 5 | 16.6666667 | 36.67 |
|  |  | 10 |  | 30 | 21 | 70 | 11 | 36.6666667 | 53.33 |
|  |  | 25 |  | 30 | 12 | 40 | 11 | 36.6666667 | 38.33 |
|  |  | 50 |  | 30 | 5 | 16.66666667 | 2 | 6.66666667 | 11.67 |
|  |  |  | 0.05 | 30 | 17 | 56.66666667 | 10 | 33.3333333 | 45.00 |
|  |  |  | 0.1 | 30 | 21 | 70 | 10 | 33.3333333 | 51.67 |
|  |  |  | 0.2 | 30 | 13 | 43.33333333 | 12 | 40 | 41.67 |
|  |  |  | 0.25 | 30 | 13 | 43.33333333 | 12 | 40 | 41.67 |
| Without Co-incubation | 0 | 5 |  | 30 | 15 | 50 | 10 | 33.3333333 | 41.67 |
|  |  | 7.5 |  | 30 | 21 | 70 | 10 | 33.3333333 | 51.67 |
|  |  | 10 |  | 30 | 21 | 70 | 11 | 36.6666667 | 53.33 |
|  |  | 25 |  | 30 | 17 | 56.66666667 | 10 | 33.3333333 | 45.00 |
|  |  | 50 |  | 30 | 15 | 50 | 12 | 40 | 45.00 |
|  |  |  | 0.05 | 30 | 20 | 66.66666667 | 10 | 33.3333333 | 50.00 |
|  |  |  | 0.1 | 30 | 20 | 66.66666667 | 19 | 63.3333333 | 65.00 |
|  |  |  | 0.2 | 30 | 15 | 50 | 20 | 66.6666667 | 58.33 |
|  |  |  | 0.25 | 30 | 18 | 60 | 13 | 43.3333333 | 51.67 |
|  | 0.5 | 5 |  | 30 | 19 | 63.33333333 | 23 | 76.6666667 | 70.00 |
|  |  | 7.5 |  | 30 | 22 | 73.33333333 | 21 | 70 | 71.67 |
|  |  | 10 |  | 30 | 27 | 90 | 21 | 70 | 80.00 |
|  |  | 25 |  | 30 | 18 | 60 | 20 | 66.6666667 | 63.33 |
|  |  | 50 |  | 30 | 10 | 33.33333333 | 3 | 10 | 21.67 |
|  |  |  | 0.05 | 30 | 24 | 80 | 27 | 90 | 85.00 |
|  |  |  | 0.1 | 30 | 19 | 63.33333333 | 27 | 90 | 76.67 |
|  |  |  | 0.2 | 30 | 16 | 53.33333333 | 23 | 76.6666667 | 65.00 |
|  |  |  | 0.25 | 30 | 17 | 56.66666667 | 26 | 86.6666667 | 71.67 |
|  | 1 | 5 |  | 30 | 25 | 83.33333333 | 12 | 40 | 61.67 |
|  |  | 7.5 |  | 30 | 20 | 66.66666667 | 10 | 33.3333333 | 50.00 |
|  |  | 10 |  | 30 | 26 | 86.66666667 | 10 | 33.3333333 | 60.00 |
|  |  | 25 |  | 30 | 21 | 70 | 10 | 33.3333333 | 51.67 |
|  |  | 50 |  | 30 | 12 | 40 | 6 | 20 | 30.00 |
|  |  |  | 0.05 | 30 | 23 | 76.66666667 | 14 | 46.6666667 | 61.67 |
|  |  |  | 0.1 | 30 | 18 | 60 | 9 | 30 | 45.00 |
|  |  |  | 0.2 | 30 | 20 | 66.66666667 | 11 | 36.6666667 | 51.67 |
|  |  |  | 0.25 | 30 | 11 | 36.66666667 | 12 | 40 | 38.33 |
