# Supplementary material for: An efficient Rhizobium rhizogenes-mediated transformation system for Cuscuta campestris
Source: PLoS One. 2025 Feb 21;20(2):e0317347. doi: 10.1371/journal.pone.0317347 (PMC11844837; doi:10.1371/journal.pone.0317347)
Supplement: S4 Table — (DOCX) [file pone.0317347.s009.docx]

**S4 Table. Raw data for Table 4**

| **Culture medium** | **BAP mg/L** | **Plate no** | **Total no of plants per plate** | **No of cuttings with tip grown on one side** | **Average tip grown on one side %** | **No of cuttings with Tip grown on both sides** | **Average tip grown on both side %** |
| --- | --- | --- | --- | --- | --- | --- | --- |
| MMS medium + 0.5mg/L NAA | 5 | 1 | 10 | 2 | 22.5 | 5 | 60 |
|  |  | 2 | 10 | 1 |  | 8 |  |
|  |  | 3 | 10 | 2 |  | 5 |  |
|  |  | 4 | 10 | 4 |  | 6 |  |
|  |  | 5 | 10 | contaminated |  |  |  |
|  |  | 6 | 10 | contaminated |  |  |  |
|  | 10 | 1 | 10 | 2 | 16.66666667 | 6 | 61.66666667 |
|  |  | 2 | 10 | 2 |  | 7 |  |
|  |  | 3 | 10 | 1 |  | 6 |  |
|  |  | 4 | 10 | 2 |  | 6 |  |
|  |  | 5 | 10 | 2 |  | 6 |  |
|  |  | 6 | 10 | 1 |  | 6 |  |
|  | 25 | 1 | 10 | 2 | 20 | 3 | 42 |
|  |  | 2 | 10 | 1 |  | 5 |  |
|  |  | 3 | 10 | 2 |  | 5 |  |
|  |  | 4 | 10 | 2 |  | 5 |  |
|  |  | 5 | 10 | 3 |  | 3 |  |
|  |  | 6 | 10 | contaminated |  |  |  |
