# Supplementary material for: An efficient Rhizobium rhizogenes-mediated transformation system for Cuscuta campestris
Source: PLoS One. 2025 Feb 21;20(2):e0317347. doi: 10.1371/journal.pone.0317347 (PMC11844837; doi:10.1371/journal.pone.0317347)
Supplement: S5 Table — (DOCX) [file pone.0317347.s010.docx]

**S5 Table. Raw data for Table 5**

| **Types of explant** | **Levels** | **Plate no** | **Cuscuta cutting marked** | **Length increase in 1st**  **3 days (mm)** | **Length increase in 2nd**  **3 days (mm)** | **Length increase in 3rd**  **3 days (mm)** | **Total growth after 9 days** | **Average growth in a plate** | **Total average** |
| --- | --- | --- | --- | --- | --- | --- | --- | --- | --- |
| Shoot tip parts harvested from wild plants (introduced to the host) | With meristem | 1 | 1 | 0.8 | contaminated | | | 8.5 | 7.308 |
|  |  |  | 2 | 2 | 2 | 0 |  |  |  |
|  |  |  | 3 | 1 | 5 | 2 | 8 |  |  |
|  |  |  | 4 | 2 | 4 | 4 | 10 |  |  |
|  |  |  | 5 | 1 | 2 | contaminated | |  |  |
|  |  |  | 6 | 0.5 | 3 | 4 | 7.5 |  |  |
|  |  | 2 | 1 | 1 | 2 | 2 | 5 | 4.625 |  |
|  |  |  | 2 | contaminated | | | |  |  |
|  |  |  | 3 | 2 | contaminated | | |  |  |
|  |  |  | 4 | 2 | 0.5 | 2 | 4.5 |  |  |
|  |  |  | 5 | 0 | 2 | 1 | 3 |  |  |
|  |  |  | 6 | 2 | 1 | 3 | 6 |  |  |
|  |  | 3 | 1 | 0 | 2 | contaminated | | 8.8 |  |
|  |  |  | 2 | 2 | 4 | 3 | 9 |  |  |
|  |  |  | 3 | 2 | 3 | 2 | 7 |  |  |
|  |  |  | 4 | 3 | 2 | 3 | 8 |  |  |
|  |  |  | 5 | 2 | 3 | 6 | 11 |  |  |
|  |  |  | 6 | 2 | 3 | 4 | 9 |  |  |
|  | Without meristem | 1 | 1 | 1 | 2 | 2.5 | 5.5 | 4.9286 | 4.998 |
|  |  |  | 2 | 0 | 0 | 0 | 0 |  |  |
|  |  |  | 3 | 0 | 0 | 0 | 0 |  |  |
|  |  |  | 4 | 2 | 3 | 3 | 8 |  |  |
|  |  |  | 5 | 3 | 2 | 2 | 7 |  |  |
|  |  |  | 6 | 1 | 3 | 3 | 7 |  |  |
|  |  |  | 7 | 2 | 2 | 3 | 7 |  |  |
|  |  | 2 | 1 | 1.5 | 1 | contaminated | | 6.6667 |  |
|  |  |  | 2 | 3 | 2 | 3 | 8 |  |  |
|  |  |  | 3 | 1 | 1 | contaminated | |  |  |
|  |  |  | 4 | 2 | 2 | 2 | 6 |  |  |
|  |  |  | 5 | 0 | 3 | 3 | 6 |  |  |
|  |  |  | 6 | 0 | 2 | contaminated | |  |  |
|  |  | 3 | 1 | 0 | 0 | 0 | 0 | 3.4 |  |
|  |  |  | 2 | 1 | 3 | 3 | 7 |  |  |
|  |  |  | 3 | 0 | 0 | 0 | 0 |  |  |
|  |  |  | 4 | 0 | 3 | 3 | 6 |  |  |
|  |  |  | 5 | 0 | 0 | 0 | 0 |  |  |
|  |  |  | 6 | 0 | 2 | 3 | 5 |  |  |
|  |  |  | 7 | 1 | 3 | 3 | 7 |  |  |
|  |  |  | 8 | 1 | 0 | 0 | 1 |  |  |
|  |  |  | 9 | 2 | 2 | 4 | 8 |  |  |
|  |  |  | 10 | 0 | 0 | 0 | 0 |  |  |
|  |  |  | 11 | 3 | contaminated | | |  |  |
|  |  |  | 12 | 2 | contaminated | | |  |  |
| Shoot tip parts from seedlings after bacteria inoculation | With host | 1 | 1 | 5 | 4 | 5 | 14 | 14.6 | 15.8 |
|  |  |  | 2 | 3 | 3 | 6 | 12 |  |  |
|  |  |  | 3 | 4 | 5 | 7 | 16 |  |  |
|  |  |  | 4 | 6 | 4 | 7 | 17 |  |  |
|  |  |  | 5 | 4 | 4 | 6 | 14 |  |  |
|  |  | 2 | 1 | 4 | 6 | 8 | 18 | 17 |  |
|  |  |  | 2 | 5 | 5 | 7 | 17 |  |  |
|  |  |  | 3 | 4 | 4 | 9 | 17 |  |  |
|  |  |  | 4 | 5 | 4 | 5 | 14 |  |  |
|  |  |  | 5 | 6 | 6 | 7 | 19 |  |  |
|  |  | 3 | 1 | 5 | 6 | 6 | 17 | 15.8 |  |
|  |  |  | 2 | 7 | 5 | 6 | 18 |  |  |
|  |  |  | 3 | 6 | 3 | 5 | 14 |  |  |
|  |  |  | 4 | 6 | 5 | 4 | 15 |  |  |
|  |  |  | 5 | 7 | 5 | 3 | 15 |  |  |
|  | Without host | 1 | 1 | 0 | 0 | 0.2 | 0.2 | 0.08 | 0.04 |
|  |  |  | 2 | 0 | 0 | 0 | 0 |  |  |
|  |  |  | 3 | 0 | 0 | 0 | 0 |  |  |
|  |  |  | 4 | 0 | 0 | 0 | 0 |  |  |
|  |  |  | 5 | 0.2 | 0 | 0 | 0.2 |  |  |
|  |  | 2 | 1 | 0 | 0 | 0 | 0 | 0.02 |  |
|  |  |  | 2 | 0 | 0 | 0 | 0 |  |  |
|  |  |  | 3 | 0 | 0 | 0.1 | 0.1 |  |  |
|  |  |  | 4 | 0 | 0 | 0 | 0 |  |  |
|  |  |  | 5 | 0 | 0 | 0 | 0 |  |  |
|  |  | 3 | 1 | 0 | 0 | 0 | 0 | 0.02 |  |
|  |  |  | 2 | 0 | 0 | 0 | 0 |  |  |
|  |  |  | 3 | 0 | 0 | 0 | 0 |  |  |
|  |  |  | 4 | 0 | 0.1 | 0 | 0.1 |  |  |
|  |  |  | 5 | 0 | 0 | 0 | 0 |  |  |
