# Supplementary material for: An efficient Rhizobium rhizogenes-mediated transformation system for Cuscuta campestris
Source: PLoS One. 2025 Feb 21;20(2):e0317347. doi: 10.1371/journal.pone.0317347 (PMC11844837; doi:10.1371/journal.pone.0317347)
Supplement: S6 Table — (Treatment 1). (DOCX) [file pone.0317347.s011.docx]

**S6 Table. Raw data for Fig 7. (Treatment 1)**

|  | Plate no | Tomato plant no | Total no of Cuscuta explants introduced | No of elongated shoots expressing YFP | YFP expression % | Average per plate |
| --- | --- | --- | --- | --- | --- | --- |
| With host | 1 | 1 | 9 | 9 | 100 |  |
|  |  | 2 | 11 | 10 | 90.909091 |  |
|  |  | 3 | 9 | 3 | 33.333333 | 74.74747 |
|  |  |  |  |  |  |  |
|  | 2 | 1 | 10 | 2 | 20 |  |
|  |  | 2 | 9 | 3 | 33.333333 |  |
|  |  | 3 | 11 | 3 | 27.272727 | 26.86869 |
|  |  |  |  |  |  |  |
|  | 3 | 1 | 8 | 3 | 37.5 |  |
|  |  | 2 | 6 | 1 | 16.666667 |  |
|  |  | 3 | 6 | 1 | 16.666667 | 23.61111 |
|  |  |  |  |  |  |  |
|  | 4 | 1 | 9 | 3 | 33.333333 |  |
|  |  | 2 | 10 | 1 | 10 |  |
|  |  | 3 | 9 | 7 | 77.777778 | 40.37037 |
|  |  |  |  |  |  |  |
|  | 5 | 1 | 7 | 1 | 14.285714 |  |
|  |  | 2 | 10 | 1 | 10 |  |
|  |  | 3 | 5 | 2 | 40 |  |
|  |  | 4 | 10 | 2 | 20 | 21.07143 |
| Without host | 1 |  | 10 | 0 | 0 |  |
|  | 2 |  | 10 | 0 | 0 |  |
|  | 3 |  | 10 | 0 | 0 |  |
