# Supplementary material for: An efficient Rhizobium rhizogenes-mediated transformation system for Cuscuta campestris
Source: PLoS One. 2025 Feb 21;20(2):e0317347. doi: 10.1371/journal.pone.0317347 (PMC11844837; doi:10.1371/journal.pone.0317347)
Supplement: S7 Table — (Treatment 2 –set 1). (DOCX) [file pone.0317347.s012.docx]

**S7 Table. Raw data for Fig 7. (Treatment 2 – set 1)**

|  | **Plate No** | **Tomato plant no** | **Total no of Cuscuta explants introduced** | **No of elongated shoots expressing YFP** | **YFP expression %** | **Average per plate** |
| --- | --- | --- | --- | --- | --- | --- |
| Transfer to the host | 1 | 1 | 4 | 3 | 75 | 62.91667 |
|  |  | 2 | 5 | 3 | 60 |  |
|  |  | 3 | 4 | 2 | 50 |  |
|  |  | 4 | 3 | 2 | 66.666667 |  |
|  |  |  |  |  |  |  |
|  | 2 | 1 | 4 | 2 | 50 | 73.33333 |
|  |  | 2 | 4 | 2 | 50 |  |
|  |  | 3 | 3 | 3 | 100 |  |
|  |  | 4 | 1 | 1 | 100 |  |
|  |  | 5 | 3 | 2 | 66.666667 |  |
|  |  |  |  |  |  |  |
|  | 3 | 1 | 2 | 1 | 50 | 37.5 |
|  |  | 2 | 3 | 2 | 66.666667 |  |
|  |  | 3 | 3 | 1 | 33.333333 |  |
|  |  | 4 | 1 | 0 | 0 |  |
|  |  |  |  |  |  |  |
|  | 4 | 1 | 7 | 6 | 85.714286 | 96.42857 |
|  |  | 2 | 4 | 4 | 100 |  |
|  |  | 3 | 2 | 2 | 100 |  |
|  |  | 4 | 2 | 2 | 100 |  |
|  |  |  |  |  |  |  |
|  | 5 | 1 | 4 | 3 | 75 | 63.33333 |
|  |  | 2 | 3 | 2 | 66.666667 |  |
|  |  | 3 | 4 | 3 | 75 |  |
|  |  | 4 | 4 | 3 | 75 |  |
|  |  | 5 | 4 | 1 | 25 |  |
|  |  |  |  |  |  |  |
|  | 6 | 1 | 4 | 3 | 75 | 75 |
|  |  | 2 | 4 | 2 | 50 |  |
|  |  | 3 | 3 | 3 | 100 |  |
|  |  | 4 | 4 | 3 | 75 |  |
|  |  |  |  |  |  |  |
|  | 7 | 1 | 3 | 3 | 100 | 78.33333 |
|  |  | 2 | 4 | 3 | 75 |  |
|  |  | 3 | 5 | 3 | 60 |  |
|  |  |  |  |  |  |  |
|  | 8 | 1 | 5 | 3 | 60 | 75.41667 |
|  |  | 2 | 4 | 3 | 75 |  |
|  |  | 3 | 3 | 2 | 66.666667 |  |
|  |  | 4 | 4 | 4 | 100 |  |
|  |  |  |  |  |  |  |
|  | 9 | 1 | 3 | 3 | 100 | 66.25 |
|  |  | 2 | 4 | 3 | 75 |  |
|  |  | 3 | 4 | 2 | 50 |  |
|  |  | 4 | 5 | 2 | 40 |  |
|  |  |  |  |  |  |  |
|  | 10 | 1 | 3 | 2 | 66.666667 | 70 |
|  |  | 2 | 5 | 3 | 60 |  |
|  |  | 3 | 6 | 5 | 83.333333 |  |
|  |  |  |  |  |  |  |
|  | 11 | 1 | 3 | 3 | 100 | 83.75 |
|  |  | 2 | 3 | 3 | 100 |  |
|  |  | 3 | 4 | 3 | 75 |  |
|  |  | 4 | 5 | 3 | 60 |  |
|  |  |  |  |  |  |  |
| no host + MMS original plates | 12 |  | 10 | 0 | 0 | 0 |
|  | 13 |  | 11 | 0 | 0 |  |
|  | 14 |  | 10 | 0 | 0 |  |
|  | 15 |  | 10 | 0 | 0 |  |
