# Supplementary material for: An efficient Rhizobium rhizogenes-mediated transformation system for Cuscuta campestris
Source: PLoS One. 2025 Feb 21;20(2):e0317347. doi: 10.1371/journal.pone.0317347 (PMC11844837; doi:10.1371/journal.pone.0317347)
Supplement: S8 Table — (Treatment 2 –set 2). (DOCX) [file pone.0317347.s013.docx]

**S8 Table. Raw data for Fig 7. (Treatment 2 – set 2)**

**T1. 0 NAA and 0 BAP, T2. 0 NAA and 10 BAP, T3. 0.5 NAA and 0 BAP, T4. 0.5 NAA and 10 BAP.**

| **Host** | **Medium** | **Treatment** | **Plate no** | **Tomato plant no** | **Total no of explants introduced** | **No of elongated shoots expressing YFP** | **YFP expression %** | **Average per plate** |
| --- | --- | --- | --- | --- | --- | --- | --- | --- |
| With host | MMS | T1 | 1 | 1 | 5 | 2 | 40 | 31.19048 |
|  |  |  |  | 2 | 4 | 1 | 25 |  |
|  |  |  |  | 3 | 7 | 2 | 28.57143 |  |
|  |  |  | 2 | 1 | 8 | 6 | 75 | 91.66667 |
|  |  |  |  | 2 | 5 | 5 | 100 |  |
|  |  |  |  | 3 | 3 | 3 | 100 |  |
|  |  | T2 | 1 | 1 | 5 | 2 | 40 | 56.66667 |
|  |  |  |  | 2 | 6 | 4 | 66.66667 |  |
|  |  |  |  | 3 | 5 | 2 | 40 |  |
|  |  |  |  | 4 | 5 | 4 | 80 |  |
|  |  |  | 2 | 1 | 5 | 1 | 20 | 53.33333 |
|  |  |  |  | 2 | 6 | 5 | 83.33333 |  |
|  |  |  |  | 3 | 5 | 3 | 60 |  |
|  |  |  |  | 4 | 4 | 2 | 50 |  |
|  |  | T3 | 1 | 1 | 3 | 3 | 100 | 77.77778 |
|  |  |  |  | 2 | 3 | 2 | 66.66667 |  |
|  |  |  |  | 3 | 3 | 2 | 66.66667 |  |
|  |  |  | 2 | 1 | 4 | 1 | 25 | 63.88889 |
|  |  |  |  | 2 | 2 | 2 | 100 |  |
|  |  |  |  | 3 | 6 | 4 | 66.66667 |  |
|  |  | T4 | 1 | 1 | 5 | 4 | 80 | 82.5 |
|  |  |  |  | 2 | 8 | 7 | 87.5 |  |
|  |  |  |  | 3 | 5 | 4 | 80 |  |
|  |  |  | 2 | 1 | 4 | 4 | 100 | 100 |
|  |  |  |  | 2 | 2 | 2 | 100 |  |
|  |  |  |  | 3 | 4 | 4 | 100 |  |
|  | 1/2 MS | T1 | 1 | 1 | 2 | 2 | 100 | 88.88889 |
|  |  |  |  | 2 | 3 | 2 | 66.66667 |  |
|  |  |  |  | 3 | 2 | 2 | 100 |  |
|  |  |  | 2 | 1 | 3 | 3 | 100 | 93.33333 |
|  |  |  |  | 2 | 2 | 2 | 100 |  |
|  |  |  |  | 3 | 5 | 4 | 80 |  |
|  |  | T2 | 1 | 1 | 3 | 1 | 33.33333 | 33.33333 |
|  |  |  |  | 2 | 3 | 1 | 33.33333 |  |
|  |  |  | 2 | 1 | 3 | 2 | 66.66667 | 88.88889 |
|  |  |  |  | 2 | 3 | 3 | 100 |  |
|  |  |  |  | 3 | 3 | 3 | 100 |  |
|  |  | T3 | 1 | 1 | 4 | 4 | 100 | 100 |
|  |  |  |  | 2 | 2 | 2 | 100 |  |
|  |  |  |  | 3 | 2 | 2 | 100 |  |
|  |  |  | 2 | 1 | 4 | 3 | 75 | 80.55556 |
|  |  |  |  | 2 | 3 | 2 | 66.66667 |  |
|  |  |  |  | 3 | 2 | 2 | 100 |  |
|  |  | T4 | 1 | 1 | 5 | 5 | 100 | 91.66667 |
|  |  |  |  | 2 | 4 | 4 | 100 |  |
|  |  |  |  | 3 | 3 | 3 | 100 |  |
|  |  |  |  | 4 | 3 | 2 | 66.66667 |  |
|  |  |  | 2 | 1 | 4 | 4 | 100 | 100 |
|  |  |  |  | 2 | 3 | 3 | 100 |  |
|  |  |  |  | 3 | 4 | 4 | 100 |  |
| Without host | MMS | T1 | 1 | 1 | 10 | 0 | 0 | 0 |
|  |  |  |  | 2 | 8 | 0 | 0 |  |
|  |  |  |  | 3 | 10 | 0 | 0 |  |
|  |  | T2 | 1 | 1 | 11 | 0 | 0 | 0 |
|  |  |  |  | 2 | 10 | 0 | 0 |  |
|  |  |  |  | 3 | 10 | 0 | 0 |  |
|  |  | T3 | 1 | 1 | 10 | 0 | 0 | 0 |
|  |  |  |  | 2 | 9 | 0 | 0 |  |
|  |  |  |  | 3 | 10 | 0 | 0 |  |
|  |  | T4 | 1 | 1 | 11 | 0 | 0 | 0 |
|  |  |  |  | 2 | 9 | 0 | 0 |  |
|  |  |  |  | 3 | 8 | 0 | 0 |  |
|  | 1/2 MS | T1 | 1 | 1 | 11 | 0 | 0 | 0 |
|  |  |  |  | 2 | 10 | 0 | 0 |  |
|  |  |  |  | 3 | 10 | 0 | 0 |  |
|  |  | T2 | 1 | 1 | 9 | 0 | 0 | 0 |
|  |  |  |  | 2 | 10 | 0 | 0 |  |
|  |  |  |  | 3 | 9 | 0 | 0 |  |
|  |  | T3 | 1 | 1 | 8 | 0 | 0 | 0 |
|  |  |  |  | 2 | 10 | 0 | 0 |  |
|  |  |  |  | 3 | 10 | 0 | 0 |  |
|  |  | T4 | 1 | 1 | 9 | 0 | 0 | 0 |
|  |  |  |  | 2 | 10 | 0 | 0 |  |
|  |  |  |  | 3 | 10 | 0 | 0 |  |
