# Supplementary material for: An efficient Rhizobium rhizogenes-mediated transformation system for Cuscuta campestris
Source: PLoS One. 2025 Feb 21;20(2):e0317347. doi: 10.1371/journal.pone.0317347 (PMC11844837; doi:10.1371/journal.pone.0317347)
Supplement: S9 Table — (Treatment 2 –set 2). (DOCX) [file pone.0317347.s014.docx]

**S9 Table. Raw data for Fig 9. (Treatment 2 – set 2)**

| **Host** | **Medium** | **Treatment** | **Plate no** | **Tomato plant no** | **Total Cuscuta explants introduced** | **Branched count** | **Branched %** | **Average per plate** |
| --- | --- | --- | --- | --- | --- | --- | --- | --- |
| With host | MMS | T1 | 1 | 1 | 6 | 1 | 16.66667 | 36.031746 |
|  |  |  |  | 2 | 5 | 1 | 20 |  |
|  |  |  |  | 3 | 7 | 5 | 71.42857 |  |
|  |  |  | 2 | 1 | 6 | 3 | 50 | 66.666667 |
|  |  |  |  | 2 | 4 | 2 | 50 |  |
|  |  |  |  | 3 | 3 | 3 | 100 |  |
|  |  | T2 | 1 | 1 | 6 | 4 | 66.66667 | 59.52381 |
|  |  |  |  | 2 | 6 | 2 | 33.33333 |  |
|  |  |  |  | 3 | 7 | 5 | 71.42857 |  |
|  |  |  |  | 4 | 6 | 4 | 66.66667 |  |
|  |  |  | 2 | 1 | 4 | 2 | 50 | 54.583333 |
|  |  |  |  | 2 | 6 | 2 | 33.33333 |  |
|  |  |  |  | 3 | 5 | 3 | 60 |  |
|  |  |  |  | 4 | 4 | 3 | 75 |  |
|  |  | T3 | 1 | 1 | 4 | 1 | 25 | 38.333333 |
|  |  |  |  | 2 | 4 | 2 | 50 |  |
|  |  |  |  | 3 | 5 | 2 | 40 |  |
|  |  |  | 2 | 1 | 2 | 1 | 50 | 38.888889 |
|  |  |  |  | 2 | 3 | 1 | 33.33333 |  |
|  |  |  |  | 3 | 3 | 1 | 33.33333 |  |
|  |  | T4 | 1 | 1 | 6 | 5 | 83.33333 | 66.825397 |
|  |  |  |  | 2 | 7 | 4 | 57.14286 |  |
|  |  |  |  | 3 | 5 | 3 | 60 |  |
|  |  |  |  |  |  |  |  | 55.714286 |
|  |  |  | 2 | 1 | 7 | 4 | 57.14286 |  |
|  |  |  |  | 2 | 5 | 3 | 60 |  |
|  |  |  |  | 3 | 4 | 2 | 50 |  |
|  | 1/2 MS | T1 | 1 | 1 | 3 | 2 | 66.66667 | 66.666667 |
|  |  |  |  | 2 | 2 | 2 | 100 |  |
|  |  |  |  | 3 | 3 | 1 | 33.33333 |  |
|  |  |  | 2 | 1 | 2 | 1 | 50 | 50 |
|  |  |  |  | 2 | 2 | 1 | 50 |  |
|  |  |  |  | 3 | 2 | 1 | 50 |  |
|  |  | T2 | 1 | 1 | 3 | 1 | 33.33333 | 44.444444 |
|  |  |  |  | 2 | 3 | 1 | 33.33333 |  |
|  |  |  |  | 3 | 3 | 2 | 66.66667 |  |
|  |  |  | 2 | 1 | 2 | 1 | 50 | 50 |
|  |  |  |  | 2 | 2 | 1 | 50 |  |
|  |  | T3 | 1 | 1 | 2 | 1 | 50 | 44.444444 |
|  |  |  |  | 2 | 3 | 1 | 33.33333 |  |
|  |  |  |  | 3 | 2 | 1 | 50 |  |
|  |  |  | 2 | 1 | 3 | 2 | 66.66667 | 50 |
|  |  |  |  | 2 | 3 | 1 | 33.33333 |  |
|  |  |  |  | 3 | 2 | 1 | 50 |  |
|  |  | T4 | 1 | 1 | 4 | 4 | 100 |  |
|  |  |  |  | 2 | 4 | 3 | 75 |  |
|  |  |  |  | 3 | 3 | 2 | 66.66667 |  |
|  |  |  |  | 4 | 1 | 1 | 100 | 85.416667 |
|  |  |  | 2 | 1 | 3 | 2 | 66.66667 | 38.888889 |
|  |  |  |  | 2 | 3 | 0 | 0 |  |
|  |  |  |  | 3 | 4 | 2 | 50 |  |
| Without host | MMS | T1 | 1 |  | 10 | 0 |  |  |
|  |  |  |  |  | 8 | 0 |  |  |
|  |  |  |  |  | 10 | 0 |  |  |
|  |  | T2 | 1 |  | 11 | 0 |  |  |
|  |  |  |  |  | 10 | 0 |  |  |
|  |  |  |  |  | 10 | 0 |  |  |
|  |  |  |  |  |  |  |  |  |
|  |  | T3 | 1 |  | 10 | 0 |  |  |
|  |  |  |  |  | 9 | 0 |  |  |
|  |  |  |  |  | 10 | 0 |  |  |
|  |  | T4 | 1 |  | 11 | 0 |  |  |
|  |  |  |  |  | 9 | 0 |  |  |
|  |  |  |  |  | 8 | 0 |  |  |
|  | 1/2 MS | T1 | 1 |  | 11 | 0 |  |  |
|  |  |  |  |  | 10 | 0 |  |  |
|  |  |  |  |  | 10 | 0 |  |  |
|  |  | T2 | 1 |  | 9 | 0 |  |  |
|  |  |  |  |  | 10 | 0 |  |  |
|  |  |  |  |  | 9 | 0 |  |  |
|  |  | T3 | 1 |  | 8 | 0 |  |  |
|  |  |  |  |  | 10 | 0 |  |  |
|  |  |  |  |  | 10 | 0 |  |  |
|  |  | T4 | 1 |  | 9 | 0 |  |  |
|  |  |  |  |  | 10 | 0 |  |  |
|  |  |  |  |  | 10 | 0 |  |  |
