# Supplementary material for: An efficient Rhizobium rhizogenes-mediated transformation system for Cuscuta campestris
Source: PLoS One. 2025 Feb 21;20(2):e0317347. doi: 10.1371/journal.pone.0317347 (PMC11844837; doi:10.1371/journal.pone.0317347)
Supplement: S10 Table — (Treatment 2 –set 1). (DOCX) [file pone.0317347.s015.docx]

**S10 Table. Raw data for Fig 9. (Treatment 2 – set 1)**

| **Plate no** | **Tomato plant no** | **Total Cuscuta explants introduced** | **Branched count** | **Branched %** | **Average per plate** |
| --- | --- | --- | --- | --- | --- |
| 1 | 1 | 4 | 3 | 75 | 93.75 |
|  | 2 | 5 | 5 | 100 |  |
|  | 3 | 3 | 3 | 100 |  |
|  | 4 | 3 | 3 | 100 |  |
| 2 | 1 | 3 | 2 | 66.6666667 | 93.3333333 |
|  | 2 | 4 | 4 | 100 |  |
|  | 3 | 2 | 2 | 100 |  |
|  | 4 | 1 | 1 | 100 |  |
|  | 5 | 2 | 2 | 100 |  |
| 3 | 1 | 2 | 2 | 100 | 100 |
|  | 2 | 2 | 2 | 100 |  |
|  | 3 | 3 | 3 | 100 |  |
|  | 4 | 1 | 1 | 100 |  |
| 4 | 1 | 5 | 5 | 100 | 100 |
|  | 2 | 3 | 3 | 100 |  |
|  | 3 | 3 | 3 | 100 |  |
|  | 4 | 3 | 3 | 100 |  |
| 5 | 1 | 3 | 3 | 100 | 88.3333333 |
|  | 2 | 3 | 3 | 100 |  |
|  | 3 | 3 | 3 | 100 |  |
|  | 4 | 4 | 3 | 75 |  |
|  | 5 | 3 | 2 | 66.6666667 |  |
| 6 | 1 | 3 | 2 | 66.6666667 | 85.4166667 |
|  | 2 | 4 | 4 | 100 |  |
|  | 3 | 2 | 2 | 100 |  |
|  | 4 | 4 | 3 | 75 |  |
| 7 | 1 | 4 | 2 | 50 | 66.6666667 |
|  | 2 | 6 | 5 | 83.3333333 |  |
|  | 3 | 6 | 4 | 66.6666667 |  |
| 8 | 1 | 5 | 3 | 60 | 75.4166667 |
|  | 2 | 4 | 3 | 75 |  |
|  | 3 | 3 | 2 | 66.6666667 |  |
|  |  | 4 | 4 | 100 |  |
| 9 | 1 | 2 | 2 | 100 | 81.6666667 |
|  | 2 | 5 | 4 | 80 |  |
|  | 3 | 3 | 2 | 66.6666667 |  |
|  | 4 | 5 | 4 | 80 |  |
| 10 | 1 | 5 | 4 | 80 | 85 |
|  | 2 | 4 | 3 | 75 |  |
|  | 3 | 6 | 6 | 100 |  |
| 11 | 1 | 4 | 3 | 75 | 79.1666667 |
|  | 2 | 4 | 3 | 75 |  |
|  | 3 | 6 | 5 | 83.3333333 |  |
|  | 4 | 6 | 5 | 83.3333333 |  |
| 12 | No host original MMS | 10 | 0 | 0 |  |
| 13 | No host original MMS | 11 | 0 | 0 |  |
| 14 | No host original MMS | 10 | 0 | 0 |  |
| 15 | No host original MMS | 10 | 0 | 0 |  |
