# Supplementary material for: An efficient Rhizobium rhizogenes-mediated transformation system for Cuscuta campestris
Source: PLoS One. 2025 Feb 21;20(2):e0317347. doi: 10.1371/journal.pone.0317347 (PMC11844837; doi:10.1371/journal.pone.0317347)
Supplement: S11 Table — (DOCX) [file pone.0317347.s016.docx]

**S1 Table.** **Composition of culture media.**

| Media | Components | Modifications |
| --- | --- | --- |
| K media  (Furuhashi, 1991)  pH = 5.7 – 5.8 | NH_4_NO_3_ - 300 mg/l  KNO_3_ - 800 mg/l  CaCl_2_.2H_2_O - 250 mg/l  MgSO_4_.7H_2_O - 260 mg/l  KH_2_PO_4_ - 120 mg/l  H_3_BO_3_-6.2 mg/l  MnSO_4_.4H_2_O-22.3 mg/l  ZnSO_4_.7H_2_O-8.6 mg/l  KI-0.83 mg/l  NaMO_4._2H_2_O-0.25 mg/l  CuSO_4_.5H_2_O-0.025 mg/l  CoCl_2_.6H_2_O-0.025 mg/l  Myo-inositol – 100 mg/l  Nicotinic acid – 1 mg/l  Pyridoxine hydrochloride – 1 mg/l  Thiamine hydrochloride – 10 mg/l  Kinetin – 1 mg/l  Sucrose – 3% (w/v)  Coconut water – 10% (v/v)  Agar – 0.9% (w/v) | Added 4.5 g/l phytagel instead of 0.9% (w/v) agar |
| MMS-1 medium  (Srivastava and Dwivedi, 2001)  pH = 5.7 – 5.8 | NH_4_NO_3_ - 300 mg/l  KNO_3_ - 800 mg/l  CaCl_2_.2H_2_O - 250 mg/l  MgSO_4_.7H_2_O - 260 mg/l  KH_2_PO_4_ - 120 mg/l  H_3_BO_3_-6.2 mg/l  MnSO_4_.4H_2_O-22.3 mg/l  ZnSO_4_.7H_2_O-8.6 mg/l  KI-0.83 mg/l  NaMO_4._2H_2_O-0.25 mg/l  CuSO_4_.5H_2_O-0.025 mg/l  CoCl_2_.6H_2_O-0.025 mg/l  Myo-inositol – 100 mg/l  Nicotinic acid – 1 mg/l  Pyridoxine hydrochloride – 1 mg/l Thiamine hydrochloride – 10 mg/l  NAA (Naphthalene acetic acid) – 3 mg/l  BA (Benzyladinine) – 1 mg/l  Glucose – 3% (w/v)  Agar – 0.9% (w/v) | Added 4.5 g/l phytagel instead of 0.9% (w/v) agar  Added BAP (6-Benzylaminopurine) instead of BA  Added different concentrations of NAA, BAP and TDZ for transformation and regeneration experiments |
| MGL (Mannitol-Glutamic acid:Luria-Bertani)  pH = 7 | Mannitol- 5 g/l  L-glutamic acid - 1 g/l  K_2_HPO_4_ - 0.25 g/l  NaCl - 0.10 g/l  MgSO_4_.7H_2_O - 0.10 g/l  Biotin - 0.001 g/l  Tryptone - 5 g/l  Yeast extract - 2.50 g/l  Agar - 15 g/l (For solid media) |  |
