# Supplementary material for: An efficient Rhizobium rhizogenes-mediated transformation system for Cuscuta campestris
Source: PLoS One. 2025 Feb 21;20(2):e0317347. doi: 10.1371/journal.pone.0317347 (PMC11844837; doi:10.1371/journal.pone.0317347)
Supplement: S12 Table — (DOCX) [file pone.0317347.s017.docx]

**S12 Table. GenBank Accession Numbers**

| **Sample** | **Accession Number** |
| --- | --- |
| *Rhizobium rhizogenes*, rolB, partial cds integrated into *Cuscuta campestris*. | PQ684448 |
| *Rhizobium rhizogenes*, rolB, partial cds integrated into *Cuscuta campestris*. | PQ684449 |
| *Rhizobium rhizogenes*, rolC gene, partial cds; integrated into *Cuscuta campestris*. | PQ684450 |
| *Rhizobium rhizogenes*, rolC gene, partial cds; integrated into *Cuscuta campestris*. | PQ684451 |
